# Sex hormones and risk of lung and colorectal cancers in women: a Mendelian randomization study

**DOI:** 10.1038/s41598-024-75305-4

**Published:** 2024-10-12

**Authors:** Marion Denos, Yi-Qian Sun, Ben Michael Brumpton, Yafang Li, Demetrius Albanes, Andrea Burnett-Hartman, Peter T. Campbell, Sébastien Küry, Christopher I. Li, Emily White, Jewel N. Samadder, Mark A. Jenkins, Xiao-Mei Mai

**Affiliations:** 1 Department of Public Health and Nursing, Norwegian University of Science and Technology, Trondheim, Norway; 2 Department of Clinical and Molecular Medicine, Norwegian University of Science and Technology, Trondheim, Norway; 3 Department of Pathology, Clinic of Laboratory Medicine, St. Olavs Hospital, Trondheim, Norway; 4 Center for Oral Health Services and Research Mid-Norway (TkMidt), Trondheim, Norway; 5 Department of Public Health and Nursing, K.G. Jebsen Centre for Genetic Epidemiology, Norwegian University of Science and Technology, Trondheim, Norway; 6 Department of Public Health and Nursing, HUNT Research Centre, NTNU, Norwegian University of Science and Technology, Levanger, 7600 Norway; 7 Clinic of Medicine, St. Olavs Hospital, Trondheim University Hospital, Trondheim, Norway; 8 Institute for Clinical and Translational Research, Baylor College of Medicine, Houston, TX USA; 9 Division of Cancer Epidemiology and Genetics, National Cancer Institute, National Institutes of Health, Bethesda, MD USA; 10 Institute for Health Research, Kaiser Permanente Colorado, Denver, CO USA; 11 Department of Epidemiology and Population Health, Albert Einstein College of Medicine, Bronx, NY USA; 12 Service de Génétique Médicale, Nantes Université, CHU Nantes, Nantes, 44000 France; 13 Public Health Sciences Division, Fred Hutchinson Cancer Center, Seattle, WA USA; 14 Department of Gastroenterology and Hepatology, Mayo Clinic, Scottsdale, AZ USA; 15 Centre for Epidemiology and Biostatistics, Melbourne School of Population and Global Health, The University of Melbourne, Melbourne, VIC Australia

**Keywords:** Colorectal cancer, Estradiol, HUNT, Lung cancer, Mendelian randomization, Sex hormones, Cancer epidemiology, Lung cancer, Colorectal cancer, Endocrinology, Epidemiology, Genetics research

## Abstract

The roles of sex hormones such as estradiol, testosterone, and sex hormone-binding globulin (SHBG) in the etiology of lung and colorectal cancers in women, among the most common cancers after breast cancer, are unclear. This Mendelian randomization (MR) study evaluated such potential causal associations in women of European ancestry. We used summary statistics data from genome-wide association studies on sex hormones and from the Trøndelag Health Study (HUNT) and large consortia on cancers. There was suggestive evidence of 1-standard deviation increase in total testosterone levels being associated with a lower risk of lung non-adenocarcinoma (hazard ratio 0.60, 95% confidence interval 0.37–0.98) in the HUNT Study. However, this was not confirmed by using data from a larger consortium. In general, we did not find convincing evidence to support a causal role of sex hormones on risk of lung and colorectal cancers in women of European ancestry.

**Supplementary Information:**

The online version contains supplementary material available at 10.1038/s41598-024-75305-4.

## Introduction

Lung and colorectal cancers are among the most common cancers in women^1^. Morbidity and mortality of lung cancer have decreased in men but increased among women in many developed countries^1^. Even though a great proportion of the sex difference can reflect changes in smoking habits, factors specific to women may play an important role^2^. All major histologic types of lung cancer are associated with smoking, the association being stronger for small-cell lung cancer than for lung adenocarcinoma^3^. Besides, around 20% of lung cancers in European females are not attributable to smoking, and lung adenocarcinoma is the most common histologic type among these women^4^. Unlike lung cancer, there is no single risk factor accounting for the majority of colorectal cancer cases^1^.

Sex hormones have been suggested to contribute to both cancers^5,6^. Both normal and cancerous lung and colonic cells contain estrogen receptors α and β^5,7,8^. Randomized controlled trials (RCTs) found that the use of estrogen plus progestin may confer a protective role against the development of colorectal cancer, particularly colon cancer, in postmenopausal women^9,10^, while estrogen plus progestin may increase lung cancer mortality^11^. Moreover, endogenous estrogen, such as estradiol, may stimulate cellular proliferation and promote lung tumor growth^5^. Recent prospective cohort studies conducted in UK Biobank reported no association between total testosterone and colorectal cancer in women^12–14^, whereas bioavailable testosterone was found to be a protective factor for colorectal cancer in a cohort study of postmenopausal women^13^. While most of the discussion on sex hormones and lung cancer has been focused on the role of estrogen, several studies reported the presence of androgen receptor in the lung and its role in promoting lung cancer development^15^. Conversely, a recent case-control study suggested higher levels of bioavailable testosterone to be associated with a reduced risk of lung cancer in 397 case-control pairs of postmenopausal never-smoking women^16^. However, the sample size of this study was too small to draw any convincing conclusions. Sex hormone-binding globulin (SHBG), the protein responsible for binding and transporting sex hormones in the bloodstream, influences their action in target tissues by regulating their bioavailability. Only 1–2% of sex hormones are unbound and therefore bioavailable^17^. SHBG was not associated with colorectal cancer among women in the recent meta-analysis^18^. Overall, results from conventional epidemiological studies investigating the associations of sex hormones with lung and colorectal tumorigenesis are conflicting.

Mendelian randomization (MR) is an analytical method using genetic variants as instrumental variables for an exposure to investigate a potential causal relationship between this exposure and an outcome^19^. This approach attempts to overcome limitations of conventional observational studies, such as reverse causation and confounding, by using genetic variants that are randomly distributed at conception^20^. The statistical power and precision of MR studies may be enhanced by using two-sample MR, where genetic variant-exposure and genetic variant-outcome associations are derived from independent samples and combined to obtain the causal effect of the exposure on the outcome^19,21^. Several MR analyses suggested causal effects of sex hormones on various diseases^22,23^. For instance, Schmitz et al. estimated a causal effect of high estradiol levels on increased bone mineral density in women^22^. Ruth et al. found evidence that higher testosterone had adverse effects on breast and endometrial cancers but reduced the risk of ovarian cancer, while SHBG had a protective effect on endometrial cancer^23^. However, few MR studies have investigated the role of sex hormones on risk of lung and colorectal cancers. Larsson et al. did not find an association between genetically predicted estradiol levels and risk of lung and colorectal cancers in women^24^. Two other recent MR studies reported that estradiol, total testosterone, bioavailable testosterone, and SHBG were unrelated to colorectal cancer^14,25^. Nevertheless, these MR studies either did not analyze subtypes of lung cancer or subsites of colorectal cancer or did not have access to outcomes data for women specifically.

In this study, we aimed to apply two-sample MR analysis to investigate the potential causal relationships between endogenous estradiol, bioavailable testosterone, total testosterone, SHBG and risk of lung and colorectal cancers in women of European ancestry: in The Trøndelag Health Study (HUNT) in Norway, the International Lung Cancer Consortium (ILCCO), FinnGen and three large consortia of colorectal cancer.

## Results

**Fig. 1:**
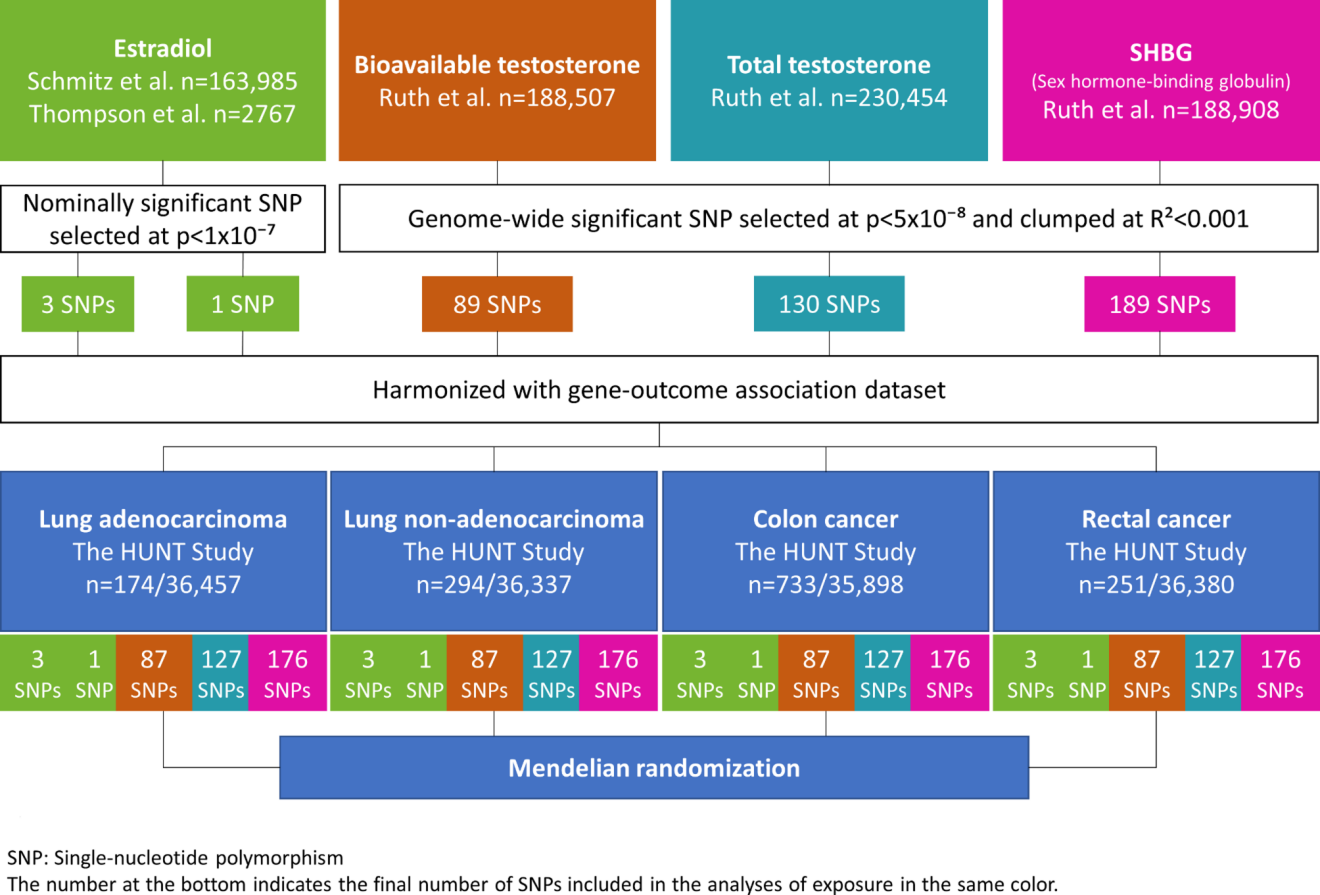
Flow chart of study methodology.

From publicly available data of genome-wide association studies (GWASs), we derived genetic instruments specific to women for sex hormones, including endogenous estradiol, bioavailable testosterone, total testosterone and SHBG. These consisted of two sets of genetic instruments for endogenous estradiol: respectively, three and one single-nucleotide polymorphisms (SNPs). The genetic instruments for bioavailable testosterone, total testosterone and SHBG levels comprised 89, 130 and 189 SNPs, respectively. Summary statistics for the associations of genetic variants of sex hormones with lung and colorectal cancers were generated in 36,631 women from the HUNT Study (Fig. 1). We performed additional MR analyses using the ILCCO, FinnGen and three large consortia of colorectal cancer data (Supplementary Fig. 1). Further details on study cohorts are provided in Supplementary Table S1^26^.

Table 1 presents the characteristics of women from the HUNT Study. Among the 36,631 women, 468 women had lung cancer including 174 lung adenocarcinoma and 294 lung non-adenocarcinoma, 733 had colon cancer and 251 rectal cancer. The mean age was 47.6 years, with 51.7% being ever smokers and 77.9% being ever passive smokers.

**Table 1:** Characteristics of women with complete information on genetic data in HUNT2 and HUNT3.

Variables	
Number of subjects	36,631
Age (years)	47.6 ± 17.1
BMI (kg/m^2^)	26.6 ± 4.7
Number of lung cancer cases (%)	468 (1.3)
Lung adenocarcinoma cases (%)	174 (0.5)
Lung non-adenocarcinoma cases (%)	294 (0.8)
Number of colorectal cancer cases (%)	984 (2.7)
Colon cases (%)	733 (2.0)
Rectal cases (%)	251 (0.7)
Smoking status, % (never/ever/unknown)	46.6/51.7/1.7
Passive smoking, % (never/ever/unknown)	21.1/77.9/1.1
Alcohol consumption, % (never/ever/unknown)	35.3/60.9/3.8
Physical activity, % (inactive/active/unknown)	21.7/49.1/29.2
Total sitting time daily (hours), % (0–7/≥8/unknown)	59.2/26.5/14.3
Family history of cancer, % (no/yes/unknown)	71.6/27.7/0.7
Reported COPD, % (no/yes)	98.1/1.9
History of diabetes, % (no/yes/unknown)	96.8/3.1/0.1

Data are given as mean ± standard deviation for continuous variables. Information on lifestyle factors were derived from questionnaires in HUNT. If women participated in both HUNT2 and HUNT3 surveys, data were retrieved from HUNT2 if available.

*BMI* body mass index, *COPD* chronic obstructive pulmonary disease.

**Fig. 2:**
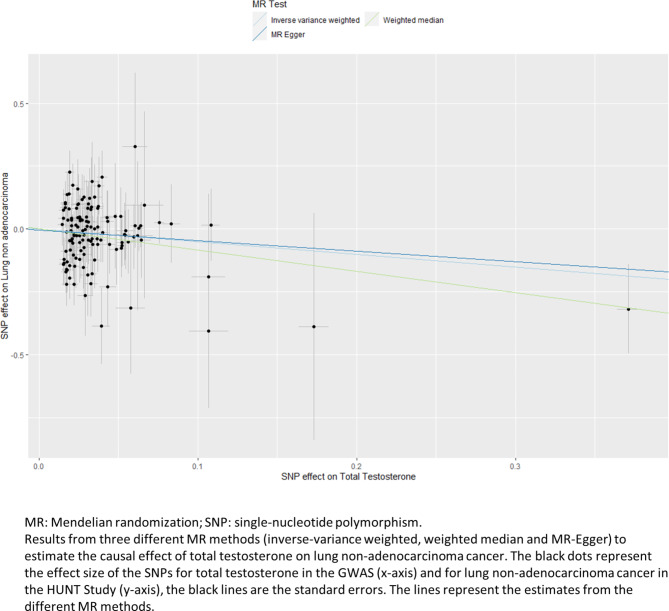
Scatter plot of genetic association between total testosterone and lung non-adenocarcinoma in HUNT.

In the MR analysis using SNP-outcome association from HUNT, the two datasets were harmonized, leaving 3 SNPs and 1 SNP for estradiol, 87 SNPs for bioavailable testosterone analyses, 127 SNPs for total testosterone analyses and 176 SNPs for SHBG analyses (Fig. 1). There were five genetic instruments in total. The genetic instruments for estradiol—proxied by 3 SNPs and 1 SNP—had a combined R^2^-value of 0.1% and 1.0% and a F-statistic of 30.3 and 28.4, respectively. The genetic instruments for bioavailable testosterone, total testosterone and SHBG had a combined R^2^-value of 4.0%, 6.4% and 12.8%, and a F-statistic of 87.6, 120.6 and 146.3, respectively. As presented in Table 2, higher estradiol levels proxied by our first genetic instrument of 3 SNPs was associated with a decreased risk of colon cancer (hazard ratio (HR) 0.38, 95% CI 0.16–0.88) based on the IVW method. Likewise, genetic predisposition to higher bioavailable testosterone was associated with a decreased risk for lung non-adenocarcinoma (HR 0.47, 95% CI 0.23–0.96, Table 3). However, the MR estimates using the weighted median method did not support the estradiol-colon cancer and bioavailable testosterone-lung non-adenocarcinoma associations, with much wider 95% CIs (HR 0.61, 95% CI 0.19–1.92 and HR 0.45, 95% CI 0.13–1.58, respectively, Table 4). Genetically predisposed higher level of total testosterone was associated with a decreased risk of lung non-adenocarcinoma (HR 0.60, 95% CI 0.37–0.98), it was supported by the weighted median method (HR 0.43, 95% CI 0.19–0.95). The MR-Egger method showed the same direction of result but did not reach statistical significance (Table 4). Figure 2 displays the scatter plot of genetic association between total testosterone and lung non-adenocarcinoma using the three methods. The result from leave-one-out analysis did not suggest that the effect of total testosterone on risk of lung non-adenocarcinoma was disproportionally influenced by a single SNP (Supplementary Fig. 2)^26^. Nevertheless, none of the above reported associations held for multiple testing (p-value = 0.05/4 (number of sex hormones) = 0.0125). The p-values for MR-Egger intercepts and Q-statistics were above 0.05 (Table 4), suggesting no strong evidence of horizontal pleiotropy or heterogeneity for the associations described.

**Table 2:** Mendelian randomization estimates for the association of estradiol level using 3-SNPs with risk of lung or colorectal cancer among women in HUNT.

	Cases	Estradiol^a^			
		HR^b^ (95% CI)	p-value	Q-statistic	p of Q-statistic
Lung cancer	468	0.78 (0.28–2.18)	0.64	10	0.008
Lung adenocarcinoma	174	0.52 (0.09–2.87)	0.45	3	0.27
Lung non-adenocarcinoma	294	1.00 (0.28–3.63)	1.00	7	0.03
Colorectal cancer	984	0.53 (0.26–1.08)	0.08	2	0.38
Colon cancer	733	0.38 (0.16–0.88)	0.02	3	0.20
Rectal cancer	251	1.32 (0.32–5.34)	0.70	2	0.43

*CI* confidence interval, *HR* hazard ratio, *IVW* inverse-variance weighted, *MR* Mendelian randomization, *SD* standard deviation.

^a^Two-sample MR was performed using data on 3-SNPs (rs4764934, rs897797, rs16991615) from Schmitz^22^ for estradiol level and from HUNT for lung or colorectal cancer.

^b^ Per one-SD increase in genetically predicted rank-transformed estradiol level, based on the IVW method.

**Table 3:** Mendelian randomization estimates for the association of bioavailable and total testosterone levels with risk of lung or colorectal cancer among women in HUNT.

	Cases	Bioavailable testosterone^a^				Total testosterone^b^			
		HR^c^ (95% CI)	p-value	Q-statistic	p of Q-statistic	HR^d^ (95% CI)	p-value	Q-statistic	p of Q-statistic
Lung cancer	468	0.64 (0.36–1.14)	0.13	61	0.98	0.72 (0.50–1.05)	0.09	103	0.94
Lung adenocarcinoma	174	1.07 (0.41–2.79)	0.88	62	0.98	0.96 (0.51–1.80)	0.91	106	0.91
Lung non-adenocarcinoma	294	0.47 (0.23–0.96)	0.04	85	0.52	0.60 (0.37–0.98)	0.04	136	0.26
Colorectal cancer	984	0.90 (0.60–1.34)	0.60	80	0.66	0.94 (0.72–1.23)	0.67	119	0.67
Colon cancer	733	0.97 (0.61–1.54)	0.88	66	0.95	0.95 (0.70–1.29)	0.73	96	0.98
Rectal cancer	251	0.68 (0.28–1.64)	0.39	105	0.08	0.89 (0.50–1.59)	0.69	158	0.03

*CI* confidence interval, *HR* hazard ratio, *IVW* inverse-variance weighted, *MR* Mendelian randomization, *SD* standard deviation.

^a^Two-sample MR was performed using summary statistics from Ruth^23^ for bioavailable testosterone level and from HUNT for lung or colorectal cancer.

^b^Two-sample MR was performed using summary statistics from Ruth^23^ for total testosterone level and from HUNT for lung or colorectal cancer.

^c^Per one-SD increase in genetically predicted bioavailable testosterone level, based on the IVW method.

^d^Per one-SD increase in genetically predicted total testosterone level, based on the IVW method.

**Table 4:** Mendelian randomization estimates and sensitivity analyses for the results in HUNT.

Method	Estradiol level^a^ on colon cancer			Bioavailable testosterone^b^ on lung non-adenocarcinoma			Total testosterone^c^ on lung non-adenocarcinoma		
	HR^d^ (95% CI)	p-value	Q-statistic/ p-value	HR^e^ (95% CI)	p-value	Q-statistic/ p-value	HR^f^ (95% CI)	p-value	Q-statistic/ p-value
IVW	0.38 (0.16–0.88)	0.02	3.25/0.20	0.47 (0.23–0.96)	0.04	84.55/0.52	0.60 (0.37–0.98)	0.04	136.62/0.26
Weighted median	0.61 (0.19–1.92)	0.40		0.45 (0.13–1.58)	0.21		0.43 (0.19–0.95)	0.04	
MR-Egger	4.73 (0.16–140.73)	0.37	1.00/0.32	0.38 (0.10–1.37)	0.14	84.40/0.50	0.66 (0.29–1.48)	0.31	135.55/0.24
MR-Egger intercept	-0.22 (-0.51–0.07)	0.13		0.008 (-0.03–0.05)	0.70		-0.004 (-0.04–0.03)	0.79	

*CI* confidence interval, *HR* hazard ratio, *IVW* inverse-variance weighted, *SD* standard deviation, *SNP* single-nucleotide polymorphism.

^a^3 SNPs/^b^87 SNPs/^c^127 SNPs were used as instrumental variables for estradiol level^22^, bioavailable and total testosterone^23^, respectively.

^d^Per one-SD increase in genetically predicted rank-transformed estradiol level.

^e^Per one-SD increase in genetically predicted bioavailable testosterone level.

^f^Per one-SD increase in genetically predicted total testosterone level.

Genetically predicted SHBG levels were not associated with the studied cancer types (Table 5). The rs727479 SNP, used as the second genetic instrument for estradiol levels, was not associated with lung and colorectal cancers (Supplementary Table S2)^26^. In addition, we found several SNPs associated with more than one sex hormone, cholesterol, body mass index, height, and alcohol consumption.

**Table 5:** Mendelian randomization estimates for the association of SHBG level with risk of lung or colorectal cancer among women in HUNT.

	Cases	SHBG^a^			
		HR^b^ (95% CI)	p-value	Q-statistic	p of Q-statistic
Lung cancer	468	1.44 (0.71–2.90)	0.31	184	0.31
Lung adenocarcinoma	174	1.04 (0.34–3.21)	0.95	177	0.44
Lung non-adenocarcinoma	294	1.73 (0.70–4.25)	0.23	190	0.20
Colorectal cancer	971	1.14 (0.70–1.84)	0.60	179	0.40
Colon cancer	723	1.26 (0.73–2.19)	0.41	175	0.49
Rectal cancer	248	0.87 (0.34–2.27)	0.78	181	0.37

*CI* confidence interval, *HR* hazard ratio, *IVW* inverse-variance weighted, *MR* Mendelian randomization, *SD* standard deviation, *SHBG* sex hormone binding globulin.

^a^Two-sample MR was performed using summary statistics from Ruth^23^ for SHBG level and from HUNT for lung or colorectal cancer.

^b^Adjusted for body mass index, per one-SD increase in genetically predicted SHBG level, based on the IVW method.

In our additional analyses using larger datasets such as ILCCO (sex-stratified), FinnGen (sex-combined) and the three colorectal cancer consortia (GECCO, CCFR and CORECT) (sex-stratified), we did not find supportive evidence for associations of sex hormones with risk of lung and colorectal cancers (Supplementary Tables S3-S5 and Supplementary Fig. 3)^26^. The only borderline association was between bioavailable testosterone and rectal cancer in the three consortia (HR 1.26, 95% CI 1.00–1.59), but the meta-analysis of the MR estimates from HUNT, FinnGen and the three consortia did not support this association (HR 1.04, 95% CI 0.76–1.41) (Supplementary Fig. 3)^26^. The p-values for the Q statistic indicated evidence of heterogeneity for a few associations such as estradiol, bioavailable or total testosterone and lung adenocarcinoma in ILCCO (Supplementary Tables S3 and S4).

## Discussion

In our two-sample MR analysis of the HUNT Study, we found a suggestive causal effect of genetically predicted higher level of total testosterone on a decreased risk of lung non-adenocarcinoma, but this was not supported by results from the larger ILCCO. Overall, our study did not provide convincing evidence for causal associations of sex hormones with risk of lung and colorectal cancers in women of European ancestry.

A limited number of MR studies have explored the potential causal associations between estradiol, bioavailable testosterone, total testosterone and SHBG, and lung and colorectal cancer risks in women^14,24,25^. Similar to the MR study of Larsson et al. investigating the effect of estradiol on lung cancer in 198,825 women in UK Biobank^24^, we did not find evidence of a causal association. To our knowledge, the only MR study investigating the causal effect of testosterone on lung cancer was performed among men^27^. In this study, Chang et al. reported no causal association of bioavailable testosterone and total testosterone with lung cancer risk in men^27^. Nevertheless, effects of sex hormones are heterogenous between males and females^22^ and may vary among lung cancer subtypes.

The recent study by Dimou et al. combined both observational analyses including 333,530 participants from the UK Biobank and MR analyses using the same GWAS for sex hormones^14^. They did not find causal associations of bioavailable testosterone and SHBG concentrations with colorectal cancer risk^14^. Although they identified a positive causal association between total testosterone and colorectal cancer in women (IVW: OR 1.09, 95% CI 1.01–1.17), this was not confirmed by their sensitivity analysis (Weighted median: OR 1.08, 95% CI 0.94–1.25)^14^. This supported the null findings in our MR study. Similarly, prospective cohort studies conducted in UK Biobank by Peila et al. and McMenamin et al. did not report any associations between total testosterone or SHBG and colorectal cancer in women^12,13^, even though McMenamin et al. found a protective effect of bioavailable testosterone on colorectal cancer in postmenopausal women^13^. Traditional observational studies may be more prone to confounding than MR studies.

A meta-analysis including four RCTs, eight cohort and eight case-control studies reported evidence of a protective role of estrogen therapy (RR: 0.79, 95% CI: 0.69–0.91) and combined estrogen-progestogen therapy (RR: 0.74, 95% CI: 0.68–0.81) on colorectal cancer^10^. Our findings did not support an association between genetically predicted estradiol levels and colorectal cancer risk in women, similarly to the MR studies of Larsson et al.^24^ and Cornish et al.^25^. The inconsistent findings in RCT and MR studies suggest that endogenous and exogenous estrogen may exert different effects on colorectal cancer risk. We also note that the genetic variants for estradiol levels are weak instruments. Future studies are needed to further investigate the role of endogenous estrogens in the prevention of colorectal cancer.

The current study is a thorough investigation of the causal associations of various sex hormones on risk of lung and colorectal cancer in women of European ancestry. The main strength of our study is the MR design, which reduced potential bias from confounders and reverse causality if the assumptions hold. These assumptions were likely satisfied by selecting genetic variants associated with bioavailable testosterone, total testosterone and SHBG at a genome-wide significance level and by relatively large F-statistics as well as applying multiple MR methods as sensitivity analyses that are more robust to pleiotropy. In addition, we explored the associations of sex hormones with lung and colorectal cancer subtypes, which were not investigated in the previous MR studies^24,25^. Finally, our study was the first to use sex-stratified genetic summary data from the ILCCO and the three colorectal cancer consortia (GECCO, CCFR and CORECT) to study such associations.

Our study had several limitations. First, the three SNPs used as instruments for estradiol in our study were nominally associated with estradiol levels at a p-value threshold of 1 × 10⁻⁷ in the UK Biobank, in which only a subset of the participants had estradiol levels above the detection limit^22^. This may result in weak instrument bias for estradiol, as also suggested by the small F-statistics for the two genetic instruments. Null associations in estradiol analyses might be due to lack of a true causal association but might also be due to weak instrument biasand insufficient statistical power to detect small effects. Second, we did not adjust for body mass index in estradiol and testosterone SNPs-outcome associations as we did for SHBG. Body mass index was not adjusted for in the original GWASs for estradiol and testosterone^22,23,28^. Two-sample MR analysis requires that the same covariates be adjusted for in both the genetic variant-exposure and genetic variant-outcome associations, and arbitrary adjustment of covariates may lead to collider bias^19^. Third, our genetic instruments included SNPs that overlapped for bioavailable testosterone, total testosterone and SHBG, and were associated with other traits, leading to potential pleiotropy effects. However, we excluded several genetic variants using LD-clumping to ensure independent variants and reduce such pleiotropy issues. In addition, there was no evidence of strong pleiotropy based on the results of Cochran’s Q and MR-Egger tests for our results in the HUNT Study, even though interpretation of MR-Egger estimate and intercept for estradiol levels should be cautious as the instrument comprised only 3 SNPs. Fourth, in our additional two-sample MR analyses, summary statistics for sex hormone SNPs-colorectal cancer associations from FinnGen were not sex-stratified. This could weaken the results as the effects of sex hormones may differ between women and men. However, meta-analysis of results from HUNT and the three colorectal consortia, with sex-specific data, would not make differences in the conclusions (data not presented). Fifth, the sample size and the number of lung and colorectal cancer cases were relatively small in the HUNT Study, making it possible to have a chance finding. To avoid this, our conclusions were drawn based on results from both the HUNT Study and the large consortia data. Finally, our analyses included women of European ancestry, limiting the generalizability of our findings to other ethnic populations.

By using summary statistics from the largest GWASs, the HUNT Study, data from ILCCO, FinnGen, three large consortia of colorectal cancer and multiple MR methods, we did not find convincing evidence for causal associations of estradiol, bioavailable testosterone, total testosterone and SHBG with lung and colorectal cancers in women of European ancestry.

## Materials and methods

### Genetic instruments

Summary statistics of sex hormones such as estradiol, bioavailable and total testosterone, SHBG were retrieved from available GWASs in women of European ancestry^22,23,28^, as presented in Supplementary Table S6^26^. For endogenous estradiol levels, we used two sets of genetic instruments. The main instrument consisted of three SNPs previously identified to be associated with estradiol in a recent GWAS conducted in the UK Biobank, including rs4764934, rs16991615 and rs10638101^22^. Four genetic variants were found to be nominally significant (p-value < 1 × 10^−7^) in this GWAS of 163,985 women^22^. Among them, rs45446698 is located close to CYP3A7, a well-known gene involved in metabolizing exogenous hormones^29^. Therefore, this SNP was not included in our first genetic instrument due to potential pleiotropic effects. The second genetic instrument for estradiol comprised the SNP rs727479 located in CYP19A1 gene. This gene encodes aromatase, an enzyme that converts androgens to estrogens in adipose tissue^30^. SNP rs727479 appeared to be nominally (p-value < 1 × 10^−7^) associated with estradiol levels in a GWAS of 2767 postmenopausal women^28^. The two genetic instruments for estradiol did not include overlapping SNPs.

Genetic instruments for bioavailable testosterone, total testosterone and SHBG levels were selected at genome-wide significant level (p-value < 5 × 10^−8^) from the largest GWAS to date, conducted in the UK Biobank^23^. To obtain independent SNPs for our genetic instrument, SNPs in linkage disequilibrium (LD) were pruned with a stricter clumping R^2^ cut-off 0.001, as performed by Hayes et al.^31^. The original GWAS identified 180 SNPs for bioavailable testosterone in 188,507 women, 254 SNPs for total testosterone in 230,454 women and 359 SNPs for SHBG in 188,908 women^23^. Following LD-clumping, the number of SNPs was then reduced to 89, 130 and 189 SNPs for bioavailable testosterone, total testosterone and SHBG, respectively^31^. Figure 1 and Supplementary Fig. 1 display the flow charts for the study methods, and Supplementary Table S1 provides further details on study cohorts^26^.

### Data sources for lung and colorectal cancers

We used data from the HUNT Study for lung and colorectal cancers. The HUNT Study is a large population-based health study in Norway^32^. The study enrolled participants aged 20 years or older in four surveys: HUNT1 (1984–1986), HUNT2 (1995–1997), HUNT3 (2006–2008) and HUNT4 (2017–2019). DNA was extracted from blood samples and stored at the HUNT Biobank. Genotyping was performed using Illumina HumanCoreExome arrays: HumanCoreExome12 v1.0 and v1.1 and UM HUNT Biobank v1.0^33^. A strict quality control was performed, and samples were excluded based on specific criteria^34^. In total, 69,716 genotype samples of European ancestry passed the quality control. Imputation was performed in two rounds, using the Haplotype Reference Consortium (HRC) and the Trans-Omics for Precision Medicine (TOPMed) reference panels^33^.

For the current study we included 36,631 women from the HUNT2 and/or HUNT3 surveys, after excluding 5313 women who did not have information on genetic variants. As the estradiol-SNP rs10638101 was not genotyped, the proxy rs897797, in perfect LD (R^2^ = 1.0) with rs10638101, was included. Using the 11-digit personal identification number for all residents, participants’ information was linked to the Cancer Registry of Norway (www.kreftregisteret.no) and diagnoses of lung and colorectal cancers were obtained up to December 31, 2018. The Tenth Revision of the International Statistical Classification of Diseases and Related Health Problems codes used for registration of lung, colon and rectal cancers are C33–C34, C18 and C19–C20, respectively. Lung cancer histologic types were classified according to the International Classification of Disease of Oncology^35^. They were further categorized into two main subtypes: adenocarcinoma and non-adenocarcinoma including all other cell types based on possible difference in etiology^4^ and the same classification in previous studies^36,37^ to increase statistical power.

Additionally, we obtained genetic summary statistics data for associations of the hormone-related variants with lung cancer in women of European ancestry from ILCCO (9332 lung cancer cases and 9118 controls)^38^. Summary data for colorectal cancer were retrieved from FinnGen (4957 colorectal cancer cases and 174,006 controls)^39^ and a meta-analysis of GWASs involving 44,117 women (20,381 colorectal cancer cases and 23,736 controls) within the Genetics and Epidemiology of Colorectal Cancer Consortium (GECCO), the Colon Cancer Family Registry (CCFR) and the Colorectal Cancer Transdisciplinary Study (CORECT) consortium^40^. We excluded the UK Biobank participants from the three colorectal cancer consortia to avoid overlap with the datasets used for estimating SNP-sex hormones associations. Sex-stratified data were available from ILCCO and the three colorectal cancer consortia but not from FinnGen. Further information on the contributing studies is presented in Supplementary Table S1^26^.

### Two-sample MR analysis

MR analysis relies on three key assumptions as follows, the instrumental variable (i) is strongly associated with the exposure (relevance assumption), (ii) is unrelated to confounding factors of the exposure-outcome relationship (independence assumption) and (iii) only affects the outcome through the exposure (exclusion restriction assumption)^19^. Here, the first two-sample MR analysis was performed using summary statistics from available GWASs for sex hormones and summary statistics from the HUNT Study for lung and colorectal cancers.

The proportion of variance in each sex hormone explained by the SNPs was estimated by a combined R^2^-value, and the strength of each instrument was assessed by the F-statistic^41^. The instrument is considered as valid if F-statistic > 10. We tested for possible pleiotropic association of SNPs with other traits, including potential confounders and mediators, at the genome-wide significance level (p-value < 5 × 10^−8^) using the Phenoscanner database and the VEP tool (https://www.ensembl.org/Tools/VEP)^42^. To obtain SNP-outcome associations from the HUNT Study, we generated coefficients (ln(HR)) and standard errors from Cox regression of risk of lung cancer overall, its subtypes (adenocarcinoma and non-adenocarcinoma), colorectal, colon and rectal cancers on each SNP using individual-level data from the HUNT2 and HUNT3 surveys. The models were adjusted for batch and 20 principal components (PCs) to account for population stratification, and additionally adjusted for body mass index for SHBG (in the SNP-outcome and SNP-exposure associations) to be consistent with the adjustment made in the original GWAS by Ruth et al.^23^. The effect estimates in the exposure and outcome datasets were harmonized to the same effect allele. We applied the inverse-variance weighted (IVW) method^41^ if the instrument consisted of multiple SNPs or Wald method if it consisted of only one SNP^43^. An IVW estimate of the causal effect combines the ratio estimates of each genetic variant in a meta-analysis model^41^. A fixed-effect IVW model assumes that each SNP is a valid instrument, while the random-effect model allows horizontal pleiotropy as long as the pleiotropy is balanced between SNPs^44^. Sensitivity analyses included weighted median method and MR-Egger method. The weighted median method can give valid MR estimates even if up to 50% of the variants are invalid^45^. The MR-Egger method gives MR estimates after taking account of pleiotropic effects. To assess presence of horizontal pleiotropy, we calculated intercept and p-value of the intercept of the MR-Egger regression^46^. We tested for heterogeneity between SNPs using Cochran’s Q statistic for the IVW and MR-Egger methods^47^. If the p-value for the Q statistic was lower than 0.05, it indicates the presence of heterogeneity and can imply the presence of pleiotropy. Scatter plots were used to visualize consistency between the different methods. Leave-one-out analyses were performed to ascertain that the effect was not driven by a single SNP.

Additionally, we ran two-sample MR analyses using summary statistics from the same GWASs for sex hormones and from large-scale consortia provided by the ILCCO (sex-stratified), FinnGen (sex-combined) and GECCO, CCFR and CORECT (sex-stratified) for lung and colorectal cancers. For colorectal, colon and rectal cancers, the two-sample MR estimates from HUNT, FinnGen and the three colorectal cancer consortia were meta-analyzed using a random-effect model to increase the statistical power of the analyses and obtain an overall estimate. We did not perform meta-analysis of the MR estimates from HUNT and ILCCO for lung cancer as the subtypes were classified differently in the two studies. Statistical analyses were performed in STATA/MP 17 (College Station, TX, USA) and R (version 4.1.3) with packages *TwoSampleMR*^48^ and *MendelianRandomization*^49^.

The study has been approved by the Regional Committees for Medical and Health Research Ethics (REK South-East 2019/337). All participants signed written informed consent on participation in HUNT, with linkage to previous HUNT surveys and specific registries in accordance with the Declaration of Helsinki. Ethical approval had also been obtained in the original studies^22,23,28,38,39^.

## Electronic supplementary material

Below is the link to the electronic supplementary material.

Supplementary Material 1

Supplementary Material 2

Supplementary Material 3

Supplementary Material 4

## Data Availability

Data from the HUNT Study that is used in research projects will, when reasonably requested by others, be made available on request to the HUNT Data Access Committee (hunt@medisin.ntnu.no). The HUNT data access information describes the policy regarding data availability (https://www.ntnu.edu/hunt/data).

## Declarations

### Disclaimer

Data from the Cancer Registry of Norway (CRN) has been used in this publication. The interpretation and reporting of these data are the sole responsibility of the authors, and no endorsement by CRN is intended nor should be inferred.
