# Supplementary material for: Sex hormones and risk of lung and colorectal cancers in women: a Mendelian randomization study
Source: Sci Rep. 2024 Oct 12;14:23891. doi: 10.1038/s41598-024-75305-4 (PMC11470916; doi:10.1038/s41598-024-75305-4)
Supplement: Supplementary file 1 — Supplementary Material 1 [file 41598_2024_75305_MOESM1_ESM.docx]

**Supplementary tables “Sex hormones and risk of lung and colorectal cancers in women: a Mendelian randomization study”**

**Supplementary Table S1.** Information on the studies and consortia from which genetic association data were obtained

| **Phenotype** | **Study or consortium** | **Ancestry** | **Sex** | **Cohort size or cases/controls** | **Units** | **Ref** |
| --- | --- | --- | --- | --- | --- | --- |
| Exposures | | | | | | |
| Estradiol | Schmitz et al. | EUR | Female | 163,985 | 1-SD of rank-transformed estradiol | (1) |
|  | Thompson et al. | EUR | Female | 2767 | Log-transformed estradiol in pmol/L | (2) |
| Bioavailable testosterone | Ruth et al. | EUR | Female | 188,507 | 1-SD bioavailable testosterone (nmol/L) | (3) |
| Total testosterone | Ruth et al. | EUR | Female | 230,454 | 1-SD total testosterone (nmol/L) | (3) |
| SHBG | Ruth et al. | EUR | Female | 188,908 | 1-SD SHBG (nmol/L) | (3) |
| Outcomes | | | | | | |
| Lung cancer | HUNT | EUR | Female | 468/36,163 | Ln(HR) for lung cancer |  |
| Colorectal cancer | HUNT | EUR | Female | 984/35,647 | Ln(HR) for colorectal cancer |  |
| Lung cancer | ILCCO | EUR | Female | 9332/9118 | Ln(HR) for lung cancer | (4) |
| Colorectal cancer | FinnGen | EUR | Both | 4957/174,006 | Ln(HR) for colorectal cancer | (5) |
| Colorectal cancer | GECCO/CCFR/CORECT | EUR | Female | 20,381/23,736 | Ln(HR) for colorectal cancer | (6) |

CCFR: Colon Cancer Family Registry; CORECT: Colorectal Cancer Transdisciplinary Study; EUR: European; GECCO: Genetics and Epidemiology of Colorectal Cancer Consortium; HUNT: The Trøndelag Health Study; ILCCO: International Lung Cancer Consortium; SD: standard deviation; SHBG: Sex hormone binding globulin.

**Supplementary Table S2.** Mendelian randomization estimates for the association of estradiol level using 1-SNP with risk of lung or colorectal cancer among women in HUNT

|  |  | rs727479¹ | |
| --- | --- | --- | --- |
|  | Cases | HR² (95% CI) | p-value |
| Lung cancer | 468 | 1.20 (0.29 – 4.91) | 0.80 |
| Lung adenocarcinoma | 174 | 2.13 (0.20 – 22.26) | 0.53 |
| Lung non-adenocarcinoma | 294 | 0.85 (0.14 – 4.98) | 0.85 |
| Colorectal cancer | 984 | 0.68 (0.26 – 1.78) | 0.43 |
| Colon cancer | 733 | 0.72 (0.23 – 2.20) | 0.56 |
| Rectal cancer | 251 | 0.59 (0.09 – 3.98) | 0.58 |

CI: confidence interval; HR: hazard ratio; MR: Mendelian randomization.

¹ Two-sample MR was performed using data on 1-SNP (rs727479) from Thompson et al. (2) for estradiol level and from HUNT for lung or colorectal cancer.

² Per one-unit increase in genetically predicted log-transformed estradiol level in pmol/L, based on the Wald method.

**Supplementary Table S3.** Mendelian randomization estimates for the association of estradiol level using 3-SNPs with risk of lung cancer among women in ILCCO

|  |  | Estradiol¹ | | | |
| --- | --- | --- | --- | --- | --- |
|  | Cases | HR² (95% CI) | p-value | Q-statistic | p of Q-statistic |
| Lung cancer | 9332 | 1.08 (0.76 – 1.52) | 0.67 | 6 | 0.04 |
| Lung adenocarcinoma | 4777 | 1.22 (0.77 – 1.94) | 0.40 | 8 | 0.02 |
| Squamous cell lung cancer | 1361 | 1.16 (0.55 – 2.43) | 0.70 | 2 | 0.42 |
| Small cell lung cancer | 862 | 1.04 (0.41 – 2.67) | 0.93 | 0.4 | 0.81 |

CI: confidence interval; HR: hazard ratio; ILCCO: International Lung Cancer Consortium; MR: Mendelian randomization; SD: standard deviation.

¹ Two-sample MR was performed using data on 3-SNPs (rs4764934, rs897797, rs16991615) from Schmitz (1) for estradiol level and from Li (4) for lung cancer.

² Per one-SD increase in genetically predicted rank-transformed estradiol level, based on the IVW method.

**Supplementary Table S4.** Mendelian randomization estimates for the association of bioavailable and total testosterone levels with risk of lung cancer among women in ILCCO

|  |  | Bioavailable testosterone¹ | | | | Total testosterone² | | | |
| --- | --- | --- | --- | --- | --- | --- | --- | --- | --- |
|  | Cases | HR³ (95% CI) | p-value | Q-statistic | p of Q-statistic | HR⁴ (95% CI) | p-value | Q-statistic | p of Q-statistic |
| Lung cancer | 9332 | 1.07 (0.87 – 1.31) | 0.54 | 105 | 0.07 | 0.99 (0.87 – 1.13) | 0.90 | 148 | 0.07 |
| Lung adenocarcinoma | 4777 | 1.04 (0.78 – 1.39) | 0.77 | 113 | 0.02 | 0.94 (0.78 – 1.13) | 0.53 | 163 | 0.01 |
| Squamous cell lung cancer | 1361 | 1.42 (0.94 – 2.12) | 0.09 | 91 | 0.31 | 1.06 (0.81 – 1.39) | 0.66 | 135 | 0.24 |
| Small cell lung cancer | 862 | 1.22 (0.74 – 2.03) | 0.43 | 86 | 0.62 | 1.00 (0.70 – 1.42) | 0.98 | 137 | 0.20 |

CI: confidence interval; HR: hazard ratio; ILCCO: International Lung Cancer Consortium; MR: Mendelian randomization; SD: standard deviation.

¹ Two-sample MR was performed using summary statistics from Ruth (86 SNPs) (3) for bioavailable testosterone level and from Li (4) for lung cancer.

² Two-sample MR was performed using summary statistics from Ruth (125 SNPs) (3) for total testosterone level and from Li (4) for lung cancer.

³ Per one-SD increase in genetically predicted bioavailable testosterone level, based on the IVW method.

⁴ Per one-SD increase in genetically predicted total testosterone level, based on the IVW method.

**Supplementary Table S5.** Mendelian randomization estimates for the association of SHBG level with risk of lung cancer among women in ILCCO

|  |  | SHBG¹ | | | |
| --- | --- | --- | --- | --- | --- |
|  | Cases | HR² (95% CI) | p-value | Q-statistic | p of Q-statistic |
| Lung cancer | 9332 | 1.01 (0.81 – 1.26) | 0.91 | 171 | 0.31 |
| Lung adenocarcinoma | 4777 | 1.03 (0.77 – 1.39) | 0.83 | 169 | 0.37 |
| Squamous cell lung cancer | 1361 | 1.08 (0.65 – 1.77) | 0.77 | 189 | 0.08 |
| Small cell lung cancer | 862 | 0.91 (0.50 – 1.66) | 0.76 | 155 | 0.66 |

CI: confidence interval; HR: hazard ratio; ILCCO: International Lung Cancer Consortium; MR: Mendelian randomization; SD: standard deviation; SHBG: sex hormone binding globulin.

¹ Two-sample MR was performed using summary statistics from Ruth (164 SNPs) (3) for SHBG level and from Li (4) for lung cancer.

² Per one-SD increase in genetically predicted SHBG level, based on the IVW method.

**Supplementary Table S6.** Characteristics of SNPs included in the analyses

| Nb | SNP | Gene/closest gene | Chr¹ | Allele: effect²/ other | Effect allele² frequency (HUNT) | Effect allele² frequency (GWAS³) | Beta (GWAS³) | Standard error (GWAS³) | p-value (GWAS³) |
| --- | --- | --- | --- | --- | --- | --- | --- | --- | --- |
| **Estradiol** | | | | | | | |  |  |
| 1 | rs4764934 | ASCL1 | 12 | C/T | 0.77 | 0.82 | 0.0848 | 0.0156 | 6.07E-08 |
| 2 | rs16991615 | MCM8 | 20 | A/G | 0.09 | 0.07 | 0.1300 | 0.0238 | 4.67E-08 |
| 3 | rs897797 | TMEM150B | 19 | T/G | 0.55 | 0.50 | 0.0677 | 0.0120 | 7.89E-08 |
| 4 | rs727479 | CYP19A1 | 15 | A/C | 0.67 | 0.66 | 0.0960 | 0.0180 | 7.40E-08 |
| **Bioavailable testosterone** | | | | | | | | | |
| 5 | rs10504255 | CYP7A1 | 8 | G/A | 0.33 | 0.34 | 0.0174 | 0.0027 | 6.10E-10 |
| 6 | rs10757893 | - | 9 | A/G | 0.54 | 0.58 | 0.0137 | 0.0026 | 9.30E-09 |
| 7 | rs10851395 | IVD | 15 | C/T | 0.55 | 0.52 | 0.0189 | 0.0025 | 1.10E-13 |
| 8 | rs11031005 | FSHB | 11 | C/T | 0.15 | 0.14 | 0.0230 | 0.0036 | 1.50E-10 |
| 9 | rs11078597 | MIR22HG | 17 | T/C | 0.81 | 0.81 | 0.0193 | 0.0032 | 5.00E-09 |
| 10 | rs1119208 | PDE8B | 5 | C/T | 0.65 | 0.65 | 0.0175 | 0.0027 | 1.20E-11 |
| 11 | rs11235688 | P2RY2 | 11 | G/A | 0.61 | 0.58 | 0.0165 | 0.0026 | 7.00E-12 |
| 12 | rs112635299 | SERPINA2 | 14 | G/T | 0.98 | 0.98 | 0.1023 | 0.0089 | 1.10E-31 |
| 13 | rs113172275 | SLC22A24 | 11 | C/T | 0.07 | 0.07 | 0.0492 | 0.0051 | 1.40E-22 |
| 14 | rs113347955 | GDF6 | 8 | G/A | 0.98 | 0.98 | 0.0446 | 0.0084 | 1.20E-08 |
| 15 | rs114165349 | LOC101928728 | 1 | C/G | 0.04 | 0.02 | 0.0931 | 0.0084 | 3.80E-28 |
| 16 | rs114303452 | HGFAC | 4 | A/G | 0.99 | 0.99 | 0.0919 | 0.0123 | 1.80E-13 |
| 17 | rs11621792 | NYNRIN | 14 | T/C | 0.46 | 0.45 | 0.0245 | 0.0026 | 1.40E-22 |
| 18 | rs11653686 | MIR6129 | 17 | C/T | 0.94 | 0.91 | 0.0657 | 0.0045 | 8.30E-48 |
| 19 | rs11683361 | MIR4435-2HG | 2 | G/C | 0.76 | 0.81 | 0.0223 | 0.0032 | 2.10E-12 |
| 20 | rs1171617 | SLC16A9 | 10 | T/G | 0.78 | 0.77 | 0.0295 | 0.0030 | 3.60E-23 |
| 21 | rs117327231 | CYB5A | 18 | A/C | 0.01 | 0.02 | 0.0972 | 0.0085 | 2.80E-31 |
| 22 | rs11784903 | LINC00536 | 8 | C/T | 0.49 | 0.53 | 0.0136 | 0.0025 | 3.60E-08 |
| 23 | rs11879227 | SNRPD2 | 19 | A/G | 0.79 | 0.82 | 0.0201 | 0.0033 | 1.00E-09 |
| 24 | rs12135478 | MACF1 | 1 | G/A | 0.3 | 0.33 | 0.0151 | 0.0027 | 8.80E-09 |
| 25 | rs1214759 | ZNF318 | 6 | G/A | 0.67 | 0.68 | 0.0181 | 0.0027 | 2.80E-12 |
| 26 | rs12189146 | PRLR | 5 | G/A | 0.94 | 0.95 | 0.0320 | 0.0058 | 1.80E-08 |
| 27 | rs12543598 | CYP11B1 | 8 | T/G | 0.43 | 0.45 | 0.0199 | 0.0025 | 6.60E-15 |
| 28 | rs12564492 | TBX19 | 1 | A/G | 0.73 | 0.7 | 0.0153 | 0.0028 | 5.00E-09 |
| 29 | rs12645584 | LCORL | 4 | T/C | 0.74 | 0.73 | 0.0169 | 0.0029 | 3.90E-09 |
| 30 | rs12658172 | ZNF608 | 5 | G/C | 0.86 | 0.84 | 0.0303 | 0.0035 | 1.80E-18 |
| 31 | rs12683780 | C9orf92 | 9 | A/C | 0.72 | 0.67 | 0.0179 | 0.0028 | 4.70E-12 |
| 32 | rs13020003 | STON1-GTF2A1L | 2 | C/A | 0.32 | 0.33 | 0.0170 | 0.0027 | 4.40E-10 |
| 33 | rs13153019 | LMAN2 | 5 | C/T | 0.28 | 0.25 | 0.0177 | 0.0029 | 2.30E-09 |
| 34 | rs149624078 | WDR72 | 15 | T/C | 0.04 | 0.01 | 0.0791 | 0.0111 | 3.10E-12 |
| 35 | rs1515098 | LOC646736 | 2 | T/C | 0.63 | 0.68 | 0.0153 | 0.0027 | 9.00E-09 |
| 36 | rs168189 | LHFPL2 | 5 | T/C | 0.3 | 0.28 | 0.0203 | 0.0028 | 5.40E-13 |
| 37 | rs1688043 | HPN | 19 | C/T | 0.05 | 0.07 | 0.0407 | 0.0051 | 1.90E-16 |
| 38 | rs17245822 | MZT1 | 13 | C/A | 0.38 | 0.37 | 0.0139 | 0.0026 | 3.60E-08 |
| 39 | rs17287714 | C4orf45 | 4 | T/C | 0.89 | 0.88 | 0.0221 | 0.0040 | 3.70E-08 |
| 40 | rs17580 | SERPINA1 | 14 | T/A | 0.97 | 0.95 | 0.0402 | 0.0059 | 6.90E-12 |
| 41 | rs1989147 | UTS2 | 1 | C/T | 0.8 | 0.81 | 0.0236 | 0.0032 | 7.00E-14 |
| 42 | rs2152318 | SOAT1 | 1 | T/C | 0.23 | 0.24 | 0.0318 | 0.0029 | 1.80E-27 |
| 43 | rs2266782 | FMO3 | 1 | A/G | 0.45 | 0.42 | 0.0161 | 0.0026 | 2.60E-11 |
| 44 | rs2374456 | LINC01819 | 2 | G/C | 0.58 | 0.58 | 0.0158 | 0.0026 | 7.40E-10 |
| 45 | rs2397112 | GSTA5 | 6 | A/G | 0.57 | 0.57 | 0.0161 | 0.0026 | 4.00E-10 |
| 46 | rs2587507 | CBX4 | 17 | C/T | 0.49 | 0.51 | 0.0145 | 0.0025 | 9.60E-09 |
| 47 | rs34269793 | MIR3681HG | 2 | C/T | 0.08 | 0.05 | 0.0333 | 0.0057 | 2.20E-10 |
| 48 | rs34931250 | ABCA8 | 17 | C/T | 0.92 | 0.94 | 0.0399 | 0.0053 | 3.10E-15 |
| 49 | rs351370 | WNT2B | 1 | T/C | 0.59 | 0.59 | 0.0162 | 0.0026 | 1.50E-10 |
| 50 | rs35783704 | - | 8 | A/G | 0.12 | 0.1 | 0.0327 | 0.0042 | 4.70E-16 |
| 51 | rs388430 | ADCY9 | 16 | C/T | 0.69 | 0.68 | 0.0167 | 0.0027 | 2.60E-10 |
| 52 | rs4149056 | SLCO1B1 | 12 | C/T | 0.17 | 0.15 | 0.0426 | 0.0035 | 3.00E-35 |
| 53 | rs4368453 | LINC00880 | 3 | T/C | 0.25 | 0.3 | 0.0171 | 0.0028 | 3.60E-09 |
| 54 | rs45446698 | CYP3A7-CYP3A51P | 7 | T/G | 0.96 | 0.96 | 0.1611 | 0.0063 | 5.10E-148 |
| 55 | rs4712976 | SLC17A3 | 6 | C/T | 0.7 | 0.73 | 0.0186 | 0.0029 | 3.30E-11 |
| 56 | rs4869893 | ARID1B | 6 | C/A | 0.27 | 0.28 | 0.0155 | 0.0028 | 1.10E-08 |
| 57 | rs56332871 | NR2F2-AS1 | 15 | C/A | 0.77 | 0.73 | 0.0299 | 0.0029 | 9.70E-27 |
| 58 | rs573833 | ATP2B2 | 3 | T/C | 0.45 | 0.46 | 0.0148 | 0.0025 | 4.80E-09 |
| 59 | rs58072681 | CMIP | 16 | C/T | 0.09 | 0.07 | 0.0611 | 0.0050 | 8.30E-36 |
| 60 | rs590097 | BCL2L11 | 2 | G/T | 0.66 | 0.65 | 0.0340 | 0.0026 | 4.90E-38 |
| 61 | rs6008259 | PPARA | 22 | G/A | 0.84 | 0.82 | 0.0208 | 0.0033 | 5.30E-11 |
| 62 | rs6020423 | LINC01270 | 20 | C/T | 0.77 | 0.76 | 0.0250 | 0.0030 | 2.30E-18 |
| 63 | rs61237993 | DCAF12 | 9 | A/G | 0.15 | 0.13 | 0.0294 | 0.0038 | 1.60E-15 |
| 64 | rs61856128 | AKR1C2 | 10 | C/A | 0.68 | 0.7 | 0.0346 | 0.0028 | 1.40E-36 |
| 65 | rs62025141 | KIAA1024 | 15 | A/G | 0.85 | 0.86 | 0.0263 | 0.0037 | 1.20E-13 |
| 66 | rs62144584 | PTRHD1 | 2 | T/C | 0.75 | 0.73 | 0.0189 | 0.0028 | 1.60E-10 |
| 67 | rs62223042 | BRWD1 | 21 | G/A | 0.41 | 0.37 | 0.0153 | 0.0026 | 1.10E-09 |
| 68 | rs62231822 | SLC6A6 | 3 | C/T | 0.91 | 0.9 | 0.0289 | 0.0043 | 8.30E-12 |
| 69 | rs62263023 | CAMP | 3 | A/T | 0.08 | 0.09 | 0.0281 | 0.0044 | 7.00E-11 |
| 70 | rs62396733 | TFEB | 6 | T/C | 0.15 | 0.14 | 0.0213 | 0.0037 | 5.70E-09 |
| 71 | rs629042 | FGF9 | 13 | C/G | 0.62 | 0.61 | 0.0284 | 0.0026 | 7.40E-29 |
| 72 | rs6486122 | ARNTL | 11 | T/C | 0.64 | 0.69 | 0.0167 | 0.0027 | 1.70E-09 |
| 73 | rs6684361 | S1PR1 | 1 | C/T | 0.35 | 0.31 | 0.0395 | 0.0028 | 5.40E-48 |
| 74 | rs6788984 | CTNNB1 | 3 | A/G | 0.86 | 0.86 | 0.0201 | 0.0036 | 2.40E-08 |
| 75 | rs6792725 | THRB | 3 | A/G | 0.33 | 0.31 | 0.0173 | 0.0028 | 5.00E-10 |
| 76 | rs687339 | MSL2 | 3 | T/C | 0.77 | 0.77 | 0.0396 | 0.0030 | 4.10E-39 |
| 77 | rs7078330 | WDR11 | 10 | T/C | 0.07 | 0.09 | 0.0313 | 0.0045 | 5.70E-13 |
| 78 | rs7089122 | FGFBP3 | 10 | T/C | 0.15 | 0.18 | 0.0244 | 0.0033 | 1.70E-14 |
| 79 | rs7183977 | BMF | 15 | C/T | 0.35 | 0.35 | 0.0286 | 0.0027 | 7.80E-27 |
| 80 | rs7248104 | INSR | 19 | G/A | 0.61 | 0.58 | 0.0157 | 0.0026 | 4.50E-10 |
| 81 | rs727428 | SHBG | 17 | T/C | 0.46 | 0.44 | 0.0948 | 0.0025 | 8.3E-309 |
| 82 | rs7314285 | CUX2 | 12 | T/G | 0.93 | 0.93 | 0.0375 | 0.0050 | 9.80E-14 |
| 83 | rs7633673 | MBNL1 | 3 | G/A | 0.64 | 0.59 | 0.0184 | 0.0026 | 2.60E-13 |
| 84 | rs76895963 | CCND2-AS1 | 12 | T/G | 0.98 | 0.98 | 0.0875 | 0.0097 | 5.50E-21 |
| 85 | rs7780066 | AKR1D1 | 7 | A/G | 0.84 | 0.79 | 0.0228 | 0.0031 | 3.10E-13 |
| 86 | rs8046391 | BCL7C | 16 | C/G | 0.28 | 0.27 | 0.0171 | 0.0028 | 8.80E-10 |
| 87 | rs8111359 | TYK2 | 19 | C/T | 0.88 | 0.9 | 0.0343 | 0.0044 | 8.40E-16 |
| 88 | rs850294 | GRAMD1B | 11 | T/C | 0.13 | 0.11 | 0.0337 | 0.0040 | 5.20E-18 |
| 89 | rs9399469 | PLAGL1 | 6 | A/T | 0.56 | 0.62 | 0.0198 | 0.0026 | 2.60E-14 |
| 90 | rs9636441 | B3GNT2 | 2 | C/T | 0.29 | 0.29 | 0.0168 | 0.0028 | 6.70E-10 |
| 91 | rs9987289 | LOC157273 | 8 | A/G | 0.12 | 0.09 | 0.0337 | 0.0044 | 5.50E-15 |
| **Total testosterone** | | | | | | | | | |
| 92 | rs10108398 | SDCBP | 8 | G/A | 0.25 | 0.28 | 0.0181 | 0.0032 | 1.10E-08 |
| 93 | rs10147094 | BCL11B | 14 | A/G | 0.59 | 0.61 | 0.0220 | 0.0029 | 6.90E-16 |
| 94 | rs10168169 | MIR4435-2HG | 2 | T/C | 0.77 | 0.81 | 0.0333 | 0.0036 | 5.10E-20 |
| 95 | rs1032388 | MCM9 | 6 | C/T | 0.78 | 0.78 | 0.0544 | 0.0034 | 2.50E-61 |
| 96 | rs10799713 | USP48 | 1 | G/C | 0.21 | 0.21 | 0.0271 | 0.0034 | 9.20E-16 |
| 97 | rs10817260 | SUSD1 | 9 | C/T | 0.82 | 0.81 | 0.0430 | 0.0036 | 9.30E-35 |
| 98 | rs10821415 | C9orf3 | 9 | C/A | 0.58 | 0.58 | 0.0146 | 0.0028 | 1.60E-08 |
| 99 | rs10865479 | ST3GAL5 | 2 | T/C | 0.71 | 0.71 | 0.0221 | 0.0031 | 2.40E-13 |
| 100 | rs10910476 | IRF2BP2 | 1 | C/T | 0.46 | 0.44 | 0.0168 | 0.0028 | 1.10E-09 |
| 101 | rs11024458 | SAAL1 | 11 | A/G | 0.72 | 0.73 | 0.0189 | 0.0032 | 2.30E-10 |
| 102 | rs11031005 | FSHB | 11 | C/T | 0.15 | 0.14 | 0.0326 | 0.004 | 7.20E-17 |
| 103 | rs11125180 | STON1-GTF2A1L | 2 | A/T | 0.91 | 0.91 | 0.0325 | 0.0049 | 5.40E-12 |
| 104 | rs111328885 | CYP2C8 | 10 | C/A | 0.91 | 0.88 | 0.0326 | 0.0043 | 1.50E-14 |
| 105 | rs11191421 | BORCS7 | 10 | C/G | 0.77 | 0.76 | 0.0374 | 0.0033 | 4.20E-31 |
| 106 | rs11191801 | SH3PXD2A | 10 | A/C | 0.72 | 0.71 | 0.0234 | 0.0031 | 8.70E-16 |
| 107 | rs1119208 | PDE8B | 5 | C/T | 0.65 | 0.65 | 0.0305 | 0.0029 | 9.80E-26 |
| 108 | rs11235688 | P2RY2 | 11 | G/A | 0.61 | 0.58 | 0.0223 | 0.0029 | 3.70E-16 |
| 109 | rs112635299 | SERPINA2 | 14 | G/T | 0.98 | 0.98 | 0.0621 | 0.0098 | 2.90E-11 |
| 110 | rs112694713 | PRLR | 5 | A/G | 0.99 | 0.99 | 0.1070 | 0.0124 | 2.40E-19 |
| 111 | rs11638521 | SRP14-AS1 | 15 | T/C | 0.35 | 0.35 | 0.064 | 0.0029 | 2.60E-110 |
| 112 | rs11697333 | LOC149950 | 20 | T/C | 0.64 | 0.67 | 0.0192 | 0.0030 | 1.80E-10 |
| 113 | rs1171617 | SLC16A9 | 10 | T/G | 0.78 | 0.77 | 0.0523 | 0.0033 | 5.50E-57 |
| 114 | rs117327231 | CYB5A | 18 | A/C | 0.01 | 0.02 | 0.1731 | 0.0094 | 6.80E-82 |
| 115 | rs11774829 | - | 8 | A/T | 0.12 | 0.1 | 0.056 | 0.0047 | 5.80E-32 |
| 116 | rs11782259 | ZNF703 | 8 | A/G | 0.92 | 0.91 | 0.0308 | 0.0049 | 1.10E-10 |
| 117 | rs11892043 | NCOA1 | 2 | A/G | 0.74 | 0.73 | 0.0216 | 0.0031 | 2.40E-11 |
| 118 | rs12078363 | TGFB2 | 1 | T/C | 0.69 | 0.68 | 0.0276 | 0.0030 | 1.00E-21 |
| 119 | rs1214761 | ZNF318 | 6 | G/A | 0.67 | 0.68 | 0.0311 | 0.0030 | 6.20E-26 |
| 120 | rs12185851 | C2CD2 | 21 | C/T | 0.22 | 0.23 | 0.0214 | 0.0033 | 3.20E-11 |
| 121 | rs1242518 | MED9 | 17 | T/C | 0.74 | 0.75 | 0.0205 | 0.0032 | 4.80E-10 |
| 122 | rs12436785 | LINC01550 | 14 | C/T | 0.38 | 0.42 | 0.0300 | 0.0028 | 2.80E-26 |
| 123 | rs12564492 | TBX19 | 1 | A/G | 0.73 | 0.7 | 0.0195 | 0.0031 | 3.80E-11 |
| 124 | rs1260326 | GCKR | 2 | C/T | 0.68 | 0.61 | 0.0366 | 0.0029 | 4.70E-39 |
| 125 | rs12658172 | ZNF608 | 5 | G/C | 0.86 | 0.84 | 0.0517 | 0.0039 | 5.80E-41 |
| 126 | rs12683780 | C9orf92 | 9 | A/C | 0.72 | 0.67 | 0.0309 | 0.0031 | 7.50E-26 |
| 127 | rs12708515 | C15orf39 | 15 | G/C | 0.37 | 0.36 | 0.0214 | 0.0029 | 2.10E-14 |
| 128 | rs1314911 | RAD51B | 14 | A/G | 0.85 | 0.86 | 0.0259 | 0.0040 | 7.20E-10 |
| 129 | rs13153019 | LMAN2 | 5 | C/T | 0.28 | 0.25 | 0.0243 | 0.0033 | 1.50E-14 |
| 130 | rs13184921 | FBN2 | 5 | T/C | 0.75 | 0.75 | 0.0255 | 0.0033 | 7.60E-16 |
| 131 | rs13229619 | MLXIPL | 7 | A/G | 0.13 | 0.13 | 0.0538 | 0.0042 | 8.30E-39 |
| 132 | rs13269725 | EYA1 | 8 | A/G | 0.93 | 0.92 | 0.0300 | 0.0052 | 1.80E-08 |
| 133 | rs1660322 | RNF19A | 8 | T/C | 0.7 | 0.69 | 0.0233 | 0.0030 | 4.50E-15 |
| 134 | rs17053931 | CDCA2 | 8 | A/G | 0.21 | 0.2 | 0.0206 | 0.0035 | 4.50E-09 |
| 135 | rs17201704 | CTNNB1 | 3 | T/C | 0.86 | 0.86 | 0.0433 | 0.0041 | 2.90E-28 |
| 136 | rs17245822 | MZT1 | 13 | C/A | 0.38 | 0.37 | 0.0284 | 0.0029 | 2.30E-23 |
| 137 | rs17764067 | STARD13 | 13 | G/A | 0.77 | 0.78 | 0.0208 | 0.0034 | 5.50E-10 |
| 138 | rs1872930 | AKR1D1 | 7 | T/C | 0.84 | 0.79 | 0.0514 | 0.0035 | 1.70E-54 |
| 139 | rs187370584 | LOC284009 | 17 | A/G | 0.99 | 0.99 | 0.0662 | 0.0124 | 1.50E-08 |
| 140 | rs1939769 | SLC22A24 | 11 | A/G | 0.07 | 0.07 | 0.0833 | 0.0057 | 1.30E-51 |
| 141 | rs2011425 | UGT1A8 | 2 | T/G | 0.91 | 0.92 | 0.0335 | 0.0052 | 6.60E-11 |
| 142 | rs2113944 | RORA | 15 | T/C | 0.22 | 0.22 | 0.0272 | 0.0034 | 2.70E-15 |
| 143 | rs2186945 | MC2R | 18 | C/T | 0.14 | 0.16 | 0.0249 | 0.0038 | 2.00E-10 |
| 144 | rs2344744 | LINC01624 | 6 | G/T | 0.44 | 0.41 | 0.0178 | 0.0028 | 1.30E-09 |
| 145 | rs2473140 | UTRN | 6 | C/T | 0.09 | 0.09 | 0.0292 | 0.0049 | 7.20E-10 |
| 146 | rs2608652 | GSTA1 | 6 | T/C | 0.53 | 0.53 | 0.0154 | 0.0028 | 1.30E-08 |
| 147 | rs2824138 | MIR99AHG | 21 | C/T | 0.18 | 0.18 | 0.0260 | 0.0036 | 1.70E-13 |
| 148 | rs28421540 | DLGAP1 | 18 | A/C | 0.65 | 0.71 | 0.0309 | 0.0031 | 1.90E-24 |
| 149 | rs28612846 | MIR148A | 7 | G/A | 0.78 | 0.74 | 0.0156 | 0.0032 | 4.70E-08 |
| 150 | rs2903385 | TET2 | 4 | A/G | 0.48 | 0.49 | 0.0246 | 0.0028 | 1.60E-18 |
| 151 | rs3136354 | MSH6 | 2 | C/T | 0.53 | 0.5 | 0.0237 | 0.0028 | 3.50E-18 |
| 152 | rs34163044 | STARD6 | 18 | A/C | 0.42 | 0.42 | 0.0167 | 0.0029 | 2.70E-09 |
| 153 | rs34269793 | MIR3681HG | 2 | C/T | 0.08 | 0.05 | 0.0645 | 0.0063 | 2.30E-25 |
| 154 | rs34931250 | ABCA8 | 17 | C/T | 0.92 | 0.94 | 0.0545 | 0.0059 | 3.20E-21 |
| 155 | rs36032941 | AKR1C2 | 10 | C/A | 0.68 | 0.7 | 0.0628 | 0.0031 | 3.00E-94 |
| 156 | rs36088520 | PGAP1 | 2 | T/C | 0.11 | 0.1 | 0.0283 | 0.0046 | 5.70E-09 |
| 157 | rs3776299 | ARHGAP26 | 5 | G/A | 0.53 | 0.55 | 0.0171 | 0.0028 | 3.60E-10 |
| 158 | rs4067 | TEX264 | 3 | G/A | 0.84 | 0.85 | 0.0243 | 0.004 | 1.70E-10 |
| 159 | rs4149056 | SLCO1B1 | 12 | C/T | 0.17 | 0.15 | 0.0290 | 0.0039 | 1.10E-14 |
| 160 | rs4245930 | LEF1 | 4 | G/A | 0.36 | 0.37 | 0.0254 | 0.0029 | 1.10E-19 |
| 161 | rs4294422 | EVI5 | 1 | G/A | 0.37 | 0.36 | 0.0194 | 0.0029 | 6.80E-11 |
| 162 | rs437115 | ADCY9 | 16 | T/C | 0.55 | 0.56 | 0.0279 | 0.0028 | 4.20E-24 |
| 163 | rs440150 | SLC22A18 | 11 | G/A | 0.1 | 0.09 | 0.0257 | 0.0049 | 2.20E-08 |
| 164 | rs4453027 | CTPS1 | 1 | G/T | 0.58 | 0.57 | 0.0226 | 0.0029 | 1.40E-15 |
| 165 | rs4464040 | KIAA1024 | 15 | C/T | 0.86 | 0.85 | 0.0446 | 0.0039 | 4.40E-32 |
| 166 | rs45446698 | CYP3A7-CYP3A51P | 7 | T/G | 0.96 | 0.96 | 0.3710 | 0.007 | 1.5E-635 |
| 167 | rs4632729 | UGT2B7 | 4 | A/G | 0.56 | 0.55 | 0.0276 | 0.0028 | 2.40E-22 |
| 168 | rs4736359 | CYP11B2 | 8 | T/G | 0.43 | 0.44 | 0.0370 | 0.0028 | 6.20E-40 |
| 169 | rs4804181 | ZNF799 | 19 | C/A | 0.18 | 0.22 | 0.0369 | 0.0034 | 1.30E-26 |
| 170 | rs4820829 | HORMAD2 | 22 | C/T | 0.98 | 0.98 | 0.0576 | 0.0093 | 4.10E-10 |
| 171 | rs487624 | SLC17A3 | 6 | C/A | 0.54 | 0.57 | 0.0193 | 0.0028 | 1.40E-12 |
| 172 | rs4943729 | RXFP2 | 13 | A/C | 0.55 | 0.52 | 0.0154 | 0.0028 | 1.10E-08 |
| 173 | rs4961485 | BNC2 | 9 | T/C | 0.94 | 0.93 | 0.0391 | 0.0056 | 8.90E-14 |
| 174 | rs505237 | PDE4B | 1 | G/A | 0.63 | 0.62 | 0.0157 | 0.0029 | 3.70E-08 |
| 175 | rs58072681 | CMIP | 16 | C/T | 0.09 | 0.07 | 0.1082 | 0.0055 | 7.90E-86 |
| 176 | rs58723250 | LOC100130691 | 2 | T/C | 0.18 | 0.2 | 0.0351 | 0.0035 | 2.20E-23 |
| 177 | rs590097 | BCL2L11 | 2 | G/T | 0.66 | 0.65 | 0.0606 | 0.0029 | 1.60E-100 |
| 178 | rs59741822 | CXCR4 | 2 | G/A | 0.95 | 0.92 | 0.0330 | 0.0052 | 7.00E-12 |
| 179 | rs6008259 | PPARA | 22 | G/A | 0.84 | 0.82 | 0.0338 | 0.0037 | 2.20E-22 |
| 180 | rs6020423 | LINC01270 | 20 | C/T | 0.77 | 0.76 | 0.0398 | 0.0033 | 1.10E-36 |
| 181 | rs6100174 | STX16-NPEPL1 | 20 | C/T | 0.56 | 0.62 | 0.0204 | 0.0029 | 1.70E-13 |
| 182 | rs61237993 | DCAF12 | 9 | A/G | 0.15 | 0.13 | 0.04 | 0.0042 | 6.90E-23 |
| 183 | rs61661087 | IVD | 15 | C/T | 0.55 | 0.52 | 0.0283 | 0.0028 | 1.60E-24 |
| 184 | rs61987429 | MAX | 14 | C/T | 0.68 | 0.65 | 0.0175 | 0.0030 | 9.20E-10 |
| 185 | rs62059839 | SHBG | 17 | T/C | 0.26 | 0.26 | 0.0325 | 0.0032 | 9.50E-24 |
| 186 | rs62231822 | SLC6A6 | 3 | C/T | 0.91 | 0.9 | 0.0489 | 0.0047 | 5.90E-26 |
| 187 | rs6684361 | S1PR1 | 1 | C/T | 0.35 | 0.31 | 0.0758 | 0.0030 | 7.40E-141 |
| 188 | rs674486 | HERC4 | 10 | C/T | 0.37 | 0.35 | 0.0176 | 0.0029 | 1.10E-08 |
| 189 | rs6904345 | ACOT13 | 6 | T/C | 0.62 | 0.63 | 0.0165 | 0.0029 | 1.90E-08 |
| 190 | rs6997799 | PINX1 | 8 | C/A | 0.24 | 0.23 | 0.0236 | 0.0033 | 5.80E-13 |
| 191 | rs7256920 | QPCTL | 19 | G/A | 0.46 | 0.52 | 0.0189 | 0.0028 | 5.50E-11 |
| 192 | rs72660136 | LINC00399 | 13 | T/C | 0.97 | 0.97 | 0.0605 | 0.0077 | 4.90E-16 |
| 193 | rs72693130 | HSD3B1 | 1 | A/G | 0.07 | 0.06 | 0.0312 | 0.0059 | 2.90E-08 |
| 194 | rs7342537 | ARHGEF40 | 14 | G/A | 0.02 | 0.02 | 0.1069 | 0.0106 | 2.80E-24 |
| 195 | rs7519368 | ST7L | 1 | T/A | 0.74 | 0.73 | 0.0334 | 0.0031 | 3.10E-25 |
| 196 | rs75217853 | FOXF2 | 6 | A/G | 0.11 | 0.1 | 0.0350 | 0.0047 | 1.30E-14 |
| 197 | rs75287599 | RUVBL2 | 19 | T/C | 0.09 | 0.08 | 0.0433 | 0.0053 | 1.70E-17 |
| 198 | rs7530117 | LAPTM5 | 1 | T/C | 0.35 | 0.37 | 0.0178 | 0.0029 | 1.30E-09 |
| 199 | rs7575635 | THADA | 2 | C/T | 0.82 | 0.81 | 0.0379 | 0.0035 | 1.40E-27 |
| 200 | rs75848431 | GRAMD1B | 11 | T/C | 0.15 | 0.15 | 0.0317 | 0.0039 | 4.20E-15 |
| 201 | rs7618363 | ATP2B2 | 3 | C/G | 0.83 | 0.84 | 0.0371 | 0.0038 | 1.20E-21 |
| 202 | rs76299412 | ETS1 | 11 | A/G | 0.14 | 0.15 | 0.0349 | 0.0039 | 2.00E-20 |
| 203 | rs7633673 | MBNL1 | 3 | G/A | 0.64 | 0.59 | 0.0256 | 0.0028 | 7.50E-20 |
| 204 | rs76830943 | UBQLN4 | 1 | T/C | 0.83 | 0.83 | 0.0242 | 0.0038 | 3.00E-10 |
| 205 | rs77822621 | FGFRL1 | 4 | T/C | 0.04 | 0.04 | 0.0480 | 0.0070 | 9.30E-14 |
| 206 | rs784420 | LHFPL2 | 5 | G/A | 0.31 | 0.29 | 0.0390 | 0.0031 | 1.20E-39 |
| 207 | rs8044588 | LRRC36 | 16 | C/G | 0.07 | 0.09 | 0.0285 | 0.0049 | 1.50E-09 |
| 208 | rs8111359 | TYK2 | 19 | C/T | 0.88 | 0.9 | 0.0598 | 0.0048 | 1.10E-35 |
| 209 | rs8126001 | RGS19 | 20 | T/C | 0.47 | 0.49 | 0.0197 | 0.0028 | 6.30E-13 |
| 210 | rs8184986 | CHEK2 | 22 | A/T | 0.87 | 0.87 | 0.0234 | 0.0042 | 2.40E-08 |
| 211 | rs837493 | NCOR2 | 12 | G/A | 0.44 | 0.45 | 0.0158 | 0.0028 | 1.10E-08 |
| 212 | rs873779 | PLEKHM3 | 2 | C/T | 0.41 | 0.37 | 0.0164 | 0.0029 | 1.80E-09 |
| 213 | rs9506725 | FGF9 | 13 | T/C | 0.65 | 0.63 | 0.0523 | 0.0029 | 4.80E-73 |
| 214 | rs9599996 | MZT1 | 13 | T/G | 0.57 | 0.6 | 0.0174 | 0.0029 | 2.10E-10 |
| 215 | rs9638084 | LINC01006 | 7 | G/A | 0.56 | 0.6 | 0.0206 | 0.0029 | 1.30E-13 |
| 216 | rs9687846 | C5ORF67 | 5 | G/A | 0.85 | 0.8 | 0.0264 | 0.0035 | 3.60E-12 |
| 217 | rs9832502 | RYBP | 3 | A/G | 0.22 | 0.25 | 0.0175 | 0.0032 | 3.80E-09 |
| 218 | rs9850919 | MECOM | 3 | C/T | 0.4 | 0.41 | 0.0201 | 0.0029 | 2.80E-13 |
| **SHBG** | | | | | | | | | |
| 219 | rs10095930 | LINC00536 | 8 | C/T | 0.44 | 0.42 | 0.0097 | 0.0013 | 1.10E-15 |
| 220 | rs10108150 | CYHR1 | 8 | A/G | 0.45 | 0.47 | 0.0087 | 0.0012 | 1.90E-15 |
| 221 | rs10153315 | ACTG1 | 17 | T/C | 0.61 | 0.58 | 0.0093 | 0.0012 | 7.50E-16 |
| 222 | rs10238028 | GS1-259H13.2 | 7 | G/A | 0.07 | 0.07 | 0.0142 | 0.0025 | 6.50E-09 |
| 223 | rs1033667 | CHEK2 | 22 | T/C | 0.3 | 0.3 | 0.0092 | 0.0014 | 2.50E-12 |
| 224 | rs10461018 | CCDC12 | 3 | T/C | 0.42 | 0.42 | 0.0106 | 0.0013 | 2.50E-19 |
| 225 | rs1047891 | CPS1 | 2 | A/C | 0.3 | 0.32 | 0.0179 | 0.0013 | 1.50E-44 |
| 226 | rs10511002 | - | 3 | A/C | 0.28 | 0.29 | 0.0081 | 0.0014 | 2.50E-09 |
| 227 | rs10883451 | ERLIN1 | 10 | C/T | 0.48 | 0.5 | 0.0088 | 0.0012 | 8.20E-11 |
| 228 | rs10893876 | ETS1 | 11 | C/T | 0.76 | 0.77 | 0.0080 | 0.0015 | 3.50E-08 |
| 229 | rs10961205 | LINC00583 | 9 | A/G | 0.62 | 0.58 | 0.0071 | 0.0013 | 4.60E-08 |
| 230 | rs11021232 | FAM76B | 11 | T/C | 0.8 | 0.82 | 0.0135 | 0.0016 | 4.00E-17 |
| 231 | rs11078597 | MIR22HG | 17 | C/T | 0.19 | 0.19 | 0.0156 | 0.0015 | 1.30E-25 |
| 232 | rs11186719 | TNKS2 | 10 | A/C | 0.52 | 0.52 | 0.0118 | 0.0012 | 9.30E-25 |
| 233 | rs112332688 | PAPPA | 9 | A/G | 0.76 | 0.77 | 0.0078 | 0.0015 | 1.30E-08 |
| 234 | rs1128249 | COBLL1 | 2 | T/G | 0.4 | 0.39 | 0.0217 | 0.0013 | 1.30E-73 |
| 235 | rs114165349 | LOC101928728 | 1 | G/C | 0.96 | 0.98 | 0.0740 | 0.0041 | 2.80E-82 |
| 236 | rs114949263 | TMEM176B | 7 | C/T | 0.12 | 0.11 | 0.0139 | 0.0020 | 2.70E-14 |
| 237 | rs11556924 | ZC3HC1 | 7 | T/C | 0.36 | 0.39 | 0.0113 | 0.0013 | 3.20E-18 |
| 238 | rs11601507 | TRIM5 | 11 | A/C | 0.09 | 0.07 | 0.0183 | 0.0024 | 3.20E-15 |
| 239 | rs11621792 | NYNRIN | 14 | C/T | 0.54 | 0.55 | 0.0261 | 0.0013 | 4.00E-102 |
| 240 | rs11641834 | BANP | 16 | C/T | 0.6 | 0.57 | 0.0104 | 0.0013 | 3.10E-17 |
| 241 | rs11664106 | EMILIN2 | 18 | T/A | 0.36 | 0.37 | 0.0084 | 0.0013 | 3.60E-10 |
| 242 | rs11666245 | ZNF573 | 19 | G/A | 0.96 | 0.95 | 0.0164 | 0.0029 | 1.80E-09 |
| 243 | rs11668201 | ZNF446 | 19 | T/A | 0.2 | 0.19 | 0.0086 | 0.0016 | 1.00E-08 |
| 244 | rs11690748 | FOXN2 | 2 | C/G | 0.58 | 0.62 | 0.0079 | 0.0013 | 9.70E-11 |
| 245 | rs11720108 | ADCY5 | 3 | T/C | 0.23 | 0.25 | 0.0081 | 0.0014 | 5.40E-11 |
| 246 | rs11738093 | ARL15 | 5 | A/G | 0.74 | 0.75 | 0.0139 | 0.0014 | 6.00E-24 |
| 247 | rs11774700 | SLC30A8 | 8 | C/T | 0.3 | 0.31 | 0.0075 | 0.0013 | 1.50E-09 |
| 248 | rs11967262 | VEGFA | 6 | C/G | 0.53 | 0.51 | 0.0110 | 0.0012 | 1.30E-20 |
| 249 | rs12138803 | DNM3 | 1 | C/T | 0.73 | 0.73 | 0.0086 | 0.0014 | 9.80E-10 |
| 250 | rs1223796 | PROX1 | 1 | G/C | 0.19 | 0.16 | 0.0137 | 0.0017 | 8.40E-19 |
| 251 | rs1229498 | CACNA2D1 | 7 | T/G | 0.26 | 0.28 | 0.0113 | 0.0014 | 2.40E-16 |
| 252 | rs12543287 | SLC20A2 | 8 | C/G | 0.38 | 0.37 | 0.0105 | 0.0013 | 1.80E-18 |
| 253 | rs12593818 | AQR | 15 | T/C | 0.27 | 0.26 | 0.0088 | 0.0014 | 2.40E-11 |
| 254 | rs1260326 | GCKR | 2 | C/T | 0.68 | 0.61 | 0.0352 | 0.0013 | 1.70E-188 |
| 255 | rs12797706 | OVOL1 | 11 | A/G | 0.23 | 0.23 | 0.0129 | 0.0015 | 1.60E-19 |
| 256 | rs12864658 | DLEU2 | 13 | T/C | 0.06 | 0.05 | 0.0187 | 0.0028 | 1.90E-12 |
| 257 | rs12906447 | LINC00924 | 15 | C/T | 0.55 | 0.55 | 0.0090 | 0.0013 | 4.30E-14 |
| 258 | rs13042148 | PXMP4 | 20 | C/T | 0.87 | 0.85 | 0.0157 | 0.0017 | 1.90E-21 |
| 259 | rs13108218 | HGFAC | 4 | A/G | 0.38 | 0.38 | 0.0235 | 0.0013 | 1.40E-83 |
| 260 | rs13150068 | MIR5705 | 4 | A/G | 0.57 | 0.56 | 0.0167 | 0.0012 | 6.20E-47 |
| 261 | rs13200245 | MANEA-AS1 | 6 | A/G | 0.84 | 0.84 | 0.0093 | 0.0017 | 2.20E-08 |
| 262 | rs13237750 | LOC730338 | 7 | C/T | 0.96 | 0.95 | 0.0163 | 0.0029 | 1.60E-09 |
| 263 | rs13303359 | OPTC | 1 | C/A | 0.51 | 0.53 | 0.0081 | 0.0012 | 5.90E-12 |
| 264 | rs13379043 | ELMSAN1 | 14 | C/T | 0.27 | 0.28 | 0.0113 | 0.0014 | 2.20E-16 |
| 265 | rs138204164 | LOC101929762 | 4 | C/G | 0.84 | 0.87 | 0.0116 | 0.0019 | 1.20E-10 |
| 266 | rs139974673 | CATSPER2P1 | 15 | T/C | 0.98 | 0.97 | 0.0537 | 0.0039 | 1.40E-49 |
| 267 | rs140302625 | ZNF652 | 17 | T/G | 0.06 | 0.09 | 0.0622 | 0.0021 | 4.20E-193 |
| 268 | rs140312320 | PITX3 | 10 | G/A | 0.94 | 0.93 | 0.0167 | 0.0025 | 1.10E-11 |
| 269 | rs1418652 | SLC45A3 | 1 | C/T | 0.4 | 0.39 | 0.0071 | 0.0013 | 3.30E-08 |
| 270 | rs150115323 | VGLL2 | 6 | G/C | 0.36 | 0.37 | 0.0064 | 0.0013 | 4.10E-09 |
| 271 | rs1530439 | ARID5B | 10 | T/G | 0.34 | 0.31 | 0.0115 | 0.0013 | 9.70E-19 |
| 272 | rs1634791 | HLA-C | 6 | A/G | 0.45 | 0.46 | 0.0118 | 0.0013 | 8.10E-23 |
| 273 | rs1650527 | EBF1 | 5 | C/T | 0.76 | 0.77 | 0.0137 | 0.0015 | 4.10E-22 |
| 274 | rs1684608 | MGRN1 | 16 | C/A | 0.84 | 0.81 | 0.0090 | 0.0016 | 1.50E-10 |
| 275 | rs16995626 | ADNP | 20 | C/T | 0.06 | 0.07 | 0.0180 | 0.0024 | 3.70E-15 |
| 276 | rs17202341 | CBLB | 3 | G/A | 0.35 | 0.35 | 0.0075 | 0.0013 | 1.30E-09 |
| 277 | rs1730862 | PRMT6 | 1 | G/A | 0.31 | 0.34 | 0.0227 | 0.0013 | 1.70E-73 |
| 278 | rs1741344 | SMOX | 20 | T/C | 0.59 | 0.63 | 0.0071 | 0.0013 | 3.40E-09 |
| 279 | rs174537 | MYRF | 11 | G/T | 0.64 | 0.65 | 0.0123 | 0.0013 | 5.50E-21 |
| 280 | rs17492269 | AUTS2 | 7 | G/A | 0.8 | 0.82 | 0.0098 | 0.0016 | 2.80E-09 |
| 281 | rs17580 | SERPINA1 | 14 | A/T | 0.03 | 0.05 | 0.0240 | 0.0029 | 8.90E-18 |
| 282 | rs1782652 | ZMIZ1 | 10 | T/A | 0.6 | 0.62 | 0.0136 | 0.0013 | 1.20E-26 |
| 283 | rs1801282 | PPARG | 3 | G/C | 0.14 | 0.12 | 0.0242 | 0.0019 | 8.90E-43 |
| 284 | rs1870927 | LIN9 | 1 | A/T | 0.64 | 0.62 | 0.0077 | 0.0013 | 2.00E-11 |
| 285 | rs198358 | NPPA-AS1 | 1 | C/T | 0.22 | 0.25 | 0.0085 | 0.0014 | 9.00E-11 |
| 286 | rs2018519 | HPN-AS1 | 19 | C/T | 0.2 | 0.18 | 0.0203 | 0.0016 | 3.40E-40 |
| 287 | rs202200760 | NR2F6 | 19 | C/G | 0.04 | 0.04 | 0.0761 | 0.0035 | 5.20E-120 |
| 288 | rs2057655 | C5orf56 | 5 | A/G | 0.19 | 0.19 | 0.0109 | 0.0016 | 2.40E-11 |
| 289 | rs2064074 | FMO3 | 1 | A/G | 0.5 | 0.53 | 0.0064 | 0.0012 | 3.60E-08 |
| 290 | rs2176040 | LOC646736 | 2 | A/G | 0.4 | 0.35 | 0.0151 | 0.0013 | 2.70E-36 |
| 291 | rs2207132 | MAFB | 20 | G/A | 0.94 | 0.97 | 0.0276 | 0.0034 | 9.30E-18 |
| 292 | rs2239222 | RGS6 | 14 | G/A | 0.37 | 0.35 | 0.0100 | 0.0013 | 2.10E-16 |
| 293 | rs2288926 | HDGFRP2 | 19 | A/G | 0.34 | 0.31 | 0.0069 | 0.0013 | 9.00E-09 |
| 294 | rs2364717 | NFE2L2 | 2 | T/C | 0.57 | 0.54 | 0.0076 | 0.0012 | 2.30E-12 |
| 295 | rs2498786 | AKT1 | 14 | C/G | 0.36 | 0.38 | 0.0110 | 0.0013 | 5.50E-19 |
| 296 | rs2525570 | NF1 | 17 | G/A | 0.55 | 0.6 | 0.0072 | 0.0012 | 1.00E-09 |
| 297 | rs275177 | C15orf54 | 15 | C/T | 0.17 | 0.15 | 0.0100 | 0.0018 | 5.50E-09 |
| 298 | rs28459049 | SP4 | 7 | C/T | 0.8 | 0.79 | 0.0090 | 0.0015 | 1.30E-10 |
| 299 | rs28636815 | FAM47E | 4 | G/A | 0.34 | 0.38 | 0.0117 | 0.0013 | 1.50E-21 |
| 300 | rs28712547 | PDGFC | 4 | G/A | 0.32 | 0.32 | 0.0096 | 0.0013 | 2.90E-14 |
| 301 | rs28925904 | GAB1 | 4 | C/T | 0.99 | 0.97 | 0.0227 | 0.0039 | 4.90E-09 |
| 302 | rs28929474 | SERPINA1 | 14 | T/C | 0.02 | 0.02 | 0.0609 | 0.0044 | 3.10E-43 |
| 303 | rs2915023 | C10orf11 | 10 | G/A | 0.91 | 0.9 | 0.0108 | 0.0021 | 3.90E-08 |
| 304 | rs2970871 | PPARGC1A | 4 | T/C | 0.46 | 0.44 | 0.0073 | 0.0012 | 3.20E-08 |
| 305 | rs3001032 | LOC102723886 | 1 | C/T | 0.35 | 0.32 | 0.0147 | 0.0013 | 3.60E-29 |
| 306 | rs34255979 | IRF2BP1 | 19 | T/C | 0.1 | 0.12 | 0.0282 | 0.0019 | 7.90E-52 |
| 307 | rs34331968 | CFH | 1 | T/C | 0.53 | 0.53 | 0.0111 | 0.0012 | 7.80E-20 |
| 308 | rs35233014 | RXRA | 9 | C/A | 0.26 | 0.25 | 0.0153 | 0.0014 | 3.80E-27 |
| 309 | rs3747207 | PNPLA3 | 22 | A/G | 0.24 | 0.21 | 0.0173 | 0.0015 | 1.00E-30 |
| 310 | rs390408 | UBE2L3 | 22 | A/G | 0.22 | 0.18 | 0.0142 | 0.0017 | 2.40E-18 |
| 311 | rs4077285 | ZNF787 | 19 | G/C | 0.9 | 0.91 | 0.0115 | 0.0021 | 1.80E-08 |
| 312 | rs4092465 | ONECUT2 | 18 | G/A | 0.62 | 0.65 | 0.0118 | 0.0013 | 3.60E-20 |
| 313 | rs4122352 | PDXDC1,RRN3 | 16 | A/G | 0.29 | 0.3 | 0.0108 | 0.0014 | 1.80E-16 |
| 314 | rs4149056 | SLCO1B1 | 12 | T/C | 0.83 | 0.85 | 0.0299 | 0.0017 | 1.50E-74 |
| 315 | rs4307773 | DIP2B | 12 | T/C | 0.39 | 0.42 | 0.0142 | 0.0012 | 5.90E-31 |
| 316 | rs4327143 | CYB5A | 18 | A/G | 0.71 | 0.71 | 0.0087 | 0.0014 | 1.70E-11 |
| 317 | rs4530527 | VGLL3 | 3 | C/A | 0.38 | 0.36 | 0.0079 | 0.0013 | 1.80E-09 |
| 318 | rs4563785 | SNX10 | 7 | G/T | 0.91 | 0.91 | 0.0145 | 0.0022 | 6.60E-12 |
| 319 | rs469721 | ZNF644 | 1 | C/T | 0.8 | 0.8 | 0.0107 | 0.0015 | 5.40E-12 |
| 320 | rs4709746 | LOC102724152 | 6 | T/C | 0.16 | 0.13 | 0.0118 | 0.0018 | 4.50E-11 |
| 321 | rs4810580 | EYA2 | 20 | T/G | 0.79 | 0.78 | 0.0096 | 0.0015 | 6.20E-10 |
| 322 | rs4871015 | CASC21 | 8 | A/G | 0.56 | 0.58 | 0.0079 | 0.0013 | 5.10E-10 |
| 323 | rs4876993 | UNQ6494 | 9 | T/C | 0.46 | 0.49 | 0.0069 | 0.0012 | 2.50E-09 |
| 324 | rs4976033 | PIK3R1 | 5 | A/G | 0.59 | 0.6 | 0.0106 | 0.0013 | 3.30E-17 |
| 325 | rs555754 | SLC22A3 | 6 | A/G | 0.47 | 0.47 | 0.0183 | 0.0012 | 1.70E-59 |
| 326 | rs56332871 | NR2F2-AS1 | 15 | A/C | 0.23 | 0.27 | 0.0387 | 0.0014 | 9.20E-188 |
| 327 | rs57158761 | IGF2BP2 | 3 | A/G | 0.59 | 0.56 | 0.0098 | 0.0012 | 9.30E-18 |
| 328 | rs5750131 | MB | 22 | G/A | 0.6 | 0.62 | 0.0068 | 0.0013 | 4.60E-08 |
| 329 | rs5753111 | RNF215 | 22 | T/C | 0.3 | 0.29 | 0.0129 | 0.0014 | 4.40E-23 |
| 330 | rs58489806 | MAU2 | 19 | C/T | 0.9 | 0.91 | 0.0121 | 0.0022 | 4.90E-09 |
| 331 | rs59774409 | FCGRT | 19 | T/C | 0.07 | 0.08 | 0.0180 | 0.0023 | 5.20E-16 |
| 332 | rs6073431 | HNF4A | 20 | T/C | 0.57 | 0.53 | 0.0168 | 0.0013 | 6.20E-43 |
| 333 | rs6088776 | MMP24 | 20 | T/C | 0.87 | 0.86 | 0.0105 | 0.0018 | 1.70E-10 |
| 334 | rs6129778 | ZHX3 | 20 | A/C | 0.18 | 0.19 | 0.0125 | 0.0016 | 1.60E-15 |
| 335 | rs61830291 | LINC01352 | 1 | C/A | 0.09 | 0.1 | 0.0115 | 0.0021 | 1.70E-08 |
| 336 | rs62128735 | GLTSCR1 | 19 | A/G | 0.67 | 0.7 | 0.0101 | 0.0014 | 1.80E-15 |
| 337 | rs62186584 | C2orf54 | 2 | C/T | 0.76 | 0.74 | 0.0080 | 0.0014 | 5.90E-09 |
| 338 | rs62271373 | LINC01214 | 3 | T/A | 0.96 | 0.94 | 0.0260 | 0.0026 | 2.60E-25 |
| 339 | rs62303689 | CLOCK | 4 | C/A | 0.88 | 0.87 | 0.0120 | 0.0019 | 3.20E-10 |
| 340 | rs62515079 | ZBTB10 | 8 | G/A | 0.01 | 0.02 | 0.0321 | 0.0045 | 9.20E-15 |
| 341 | rs62580766 | TXN | 9 | T/C | 0.22 | 0.18 | 0.0114 | 0.0016 | 7.50E-16 |
| 342 | rs6531735 | UBE2K | 4 | G/A | 0.49 | 0.49 | 0.0064 | 0.0012 | 1.30E-08 |
| 343 | rs6546096 | SERTAD2 | 2 | A/G | 0.23 | 0.26 | 0.0252 | 0.0014 | 3.50E-76 |
| 344 | rs6706 | TRIP6 | 7 | T/C | 0.21 | 0.18 | 0.0172 | 0.0016 | 2.50E-29 |
| 345 | rs6736913 | EML4 | 2 | A/G | 0.01 | 0.02 | 0.0368 | 0.0043 | 3.60E-21 |
| 346 | rs6772177 | NISCH | 3 | C/T | 0.85 | 0.83 | 0.0107 | 0.0016 | 1.60E-12 |
| 347 | rs67890964 | OSGIN1 | 16 | C/T | 0.41 | 0.37 | 0.0105 | 0.0013 | 7.00E-19 |
| 348 | rs6792725 | THRB | 3 | G/A | 0.67 | 0.69 | 0.0177 | 0.0014 | 4.50E-43 |
| 349 | rs68002561 | SV2A | 1 | G/A | 0.1 | 0.09 | 0.0125 | 0.0022 | 3.30E-10 |
| 350 | rs6803518 | TRH | 3 | T/C | 0.78 | 0.75 | 0.0071 | 0.0015 | 4.60E-08 |
| 351 | rs6831257 | LOC100507053 | 4 | G/A | 0.33 | 0.34 | 0.0080 | 0.0013 | 3.30E-11 |
| 352 | rs6860245 | LINC01184 | 5 | C/G | 0.23 | 0.25 | 0.0107 | 0.0014 | 9.60E-14 |
| 353 | rs687339 | MSL2 | 3 | C/T | 0.23 | 0.23 | 0.0308 | 0.0015 | 5.30E-104 |
| 354 | rs6879874 | RAB24 | 5 | T/A | 0.75 | 0.72 | 0.0075 | 0.0014 | 1.80E-09 |
| 355 | rs696825 | HNRNPK | 9 | T/C | 0.27 | 0.25 | 0.0241 | 0.0014 | 4.50E-63 |
| 356 | rs7250869 | PEPD | 19 | C/T | 0.68 | 0.69 | 0.0114 | 0.0013 | 2.70E-20 |
| 357 | rs72782727 | ZC3H7A | 16 | G/T | 0.77 | 0.75 | 0.0087 | 0.0014 | 2.30E-11 |
| 358 | rs72836346 | ACOXL | 2 | C/G | 0.1 | 0.08 | 0.0155 | 0.0023 | 6.10E-11 |
| 359 | rs72844546 | HN1 | 17 | C/T | 0.3 | 0.35 | 0.0103 | 0.0013 | 3.10E-18 |
| 360 | rs73036519 | MARK4 | 19 | G/C | 0.71 | 0.7 | 0.0086 | 0.0014 | 7.30E-10 |
| 361 | rs7321688 | CDC16 | 13 | C/A | 0.76 | 0.77 | 0.0086 | 0.0015 | 1.70E-09 |
| 362 | rs73670309 | C7orf50 | 7 | C/A | 0.91 | 0.89 | 0.0118 | 0.0020 | 1.10E-10 |
| 363 | rs740893 | NINJ2 | 12 | G/C | 0.23 | 0.21 | 0.0094 | 0.0015 | 5.00E-11 |
| 364 | rs74090351 | NFIA | 1 | A/G | 0.08 | 0.07 | 0.0194 | 0.0024 | 5.90E-18 |
| 365 | rs7475279 | AKR1C4 | 10 | A/C | 0.82 | 0.85 | 0.0183 | 0.0017 | 1.30E-30 |
| 366 | rs76491020 | CCDC73 | 11 | C/G | 0.09 | 0.09 | 0.0123 | 0.0022 | 1.30E-09 |
| 367 | rs76767219 | ZBTB10 | 8 | A/C | 0.03 | 0.03 | 0.0484 | 0.0034 | 2.40E-50 |
| 368 | rs76895963 | CCND2-AS1 | 12 | G/T | 0.02 | 0.02 | 0.0754 | 0.0047 | 1.30E-64 |
| 369 | rs7696472 | UGT2B15 | 4 | A/G | 0.46 | 0.47 | 0.0066 | 0.0012 | 4.50E-08 |
| 370 | rs78058190 | PRKAG3 | 2 | G/A | 0.92 | 0.95 | 0.0258 | 0.0032 | 7.10E-18 |
| 371 | rs784504 | CSRNP1 | 3 | C/G | 0.8 | 0.81 | 0.0105 | 0.0016 | 1.70E-12 |
| 372 | rs78890745 | C4orf45 | 4 | A/G | 0.1 | 0.11 | 0.0202 | 0.0020 | 2.40E-25 |
| 373 | rs79237700 | WDR72 | 15 | T/C | 0.96 | 0.96 | 0.0203 | 0.0032 | 7.70E-11 |
| 374 | rs79287178 | TNFSF10 | 3 | G/A | 0.97 | 0.97 | 0.0317 | 0.0037 | 4.00E-19 |
| 375 | rs79391862 | WDR72 | 15 | A/C | 0.96 | 0.99 | 0.0731 | 0.0053 | 1.70E-46 |
| 376 | rs799157 | MLXIPL | 7 | T/C | 0.03 | 0.04 | 0.0170 | 0.0030 | 9.00E-09 |
| 377 | rs80126506 | RXRA | 9 | A/G | 0.62 | 0.59 | 0.0075 | 0.0013 | 2.10E-10 |
| 378 | rs80235628 | WDR11 | 10 | G/A | 0.95 | 0.95 | 0.0247 | 0.0028 | 2.00E-17 |
| 379 | rs8107967 | MAP2K7 | 19 | G/A | 0.55 | 0.57 | 0.0086 | 0.0012 | 5.00E-14 |
| 380 | rs8178824 | APOH | 17 | C/T | 0.98 | 0.97 | 0.0427 | 0.0035 | 9.40E-36 |
| 381 | rs820504 | GLDC | 9 | G/A | 0.87 | 0.86 | 0.0137 | 0.0018 | 2.20E-16 |
| 382 | rs858519 | SHBG | 17 | C/T | 0.54 | 0.56 | 0.0988 | 0.0012 | 1.7E-1533 |
| 383 | rs8756 | HMGA2 | 12 | C/A | 0.53 | 0.49 | 0.0085 | 0.0012 | 1.70E-13 |
| 384 | rs892225 | SBNO2 | 19 | G/A | 0.37 | 0.38 | 0.0085 | 0.0013 | 2.00E-12 |
| 385 | rs899865 | - | 10 | T/C | 0.57 | 0.6 | 0.0069 | 0.0013 | 1.00E-08 |
| 386 | rs921153 | USP34 | 2 | A/G | 0.17 | 0.16 | 0.0094 | 0.0017 | 2.80E-10 |
| 387 | rs925098 | LCORL | 4 | G/A | 0.25 | 0.26 | 0.0088 | 0.0014 | 2.80E-09 |
| 388 | rs9366291 | LOC105374960 | 6 | C/G | 0.54 | 0.58 | 0.0063 | 0.0013 | 4.20E-08 |
| 389 | rs9426829 | ADAR | 1 | C/T | 0.49 | 0.48 | 0.0139 | 0.0012 | 1.50E-30 |
| 390 | rs9556403 | TGDS | 13 | G/A | 0.37 | 0.35 | 0.0072 | 0.0013 | 6.80E-10 |
| 391 | rs9644032 | SLC25A37 | 8 | T/G | 0.37 | 0.37 | 0.0082 | 0.0013 | 1.20E-11 |
| 392 | rs9697210 | PKN3 | 9 | G/A | 0.87 | 0.85 | 0.0155 | 0.0018 | 9.00E-20 |
| 393 | rs9872754 | MRAS | 3 | C/T | 0.85 | 0.84 | 0.0103 | 0.0017 | 9.00E-11 |
| 394 | rs9987289 | LOC157273 | 8 | G/A | 0.88 | 0.91 | 0.0216 | 0.0021 | 1.40E-26 |

SNP: single nucleotide polymorphism.

¹ Based on build 37.

² Effect allele = exposure-increasing allele.

³ GWASs used for estradiol-SNPs, bioavailable testosterone-SNPs, total testosterone-SNPs and SHBG-SNPs (1-3).

**References**

1. Schmitz D, Ek WE, Berggren E, Höglund J, Karlsson T, Johansson Å. Genome-wide Association Study of Estradiol Levels and the Causal Effect of Estradiol on Bone Mineral Density. J Clin Endocrinol Metab. 2021;106(11):e4471-e86.

2. Thompson DJ, O'Mara TA, Glubb DM, Painter JN, Cheng T, Folkerd E, et al. CYP19A1 fine-mapping and Mendelian randomization: estradiol is causal for endometrial cancer. Endocr Relat Cancer. 2016;23(2):77-91.

3. Ruth KS, Day FR, Tyrrell J, Thompson DJ, Wood AR, Mahajan A, et al. Using human genetics to understand the disease impacts of testosterone in men and women. Nat Med. 2020;26(2):252-8.

4. Li Y, Xiao X, Li J, Byun J, Cheng C, Bossé Y, et al. Genome-wide interaction analysis identified low-frequency variants with sex disparity in lung cancer risk. Hum Mol Genet. 2022;31(16):2831-43.

5. Kurki MI, Karjalainen J, Palta P, Sipilä TP, Kristiansson K, Donner KM, et al. FinnGen provides genetic insights from a well-phenotyped isolated population. Nature. 2023;613(7944):508-18.

6. Huyghe JR, Harrison TA, Bien SA, Hampel H, Figueiredo JC, Schmit SL, et al. Genetic architectures of proximal and distal colorectal cancer are partly distinct. Gut. 2021;70(7):1325-34.
