# Supplementary figures and images for: Sex hormones and risk of lung and colorectal cancers in women: a Mendelian randomization study
Source: Sci Rep. 2024 Oct 12;14:23891. doi: 10.1038/s41598-024-75305-4 (PMC11470916; doi:10.1038/s41598-024-75305-4)

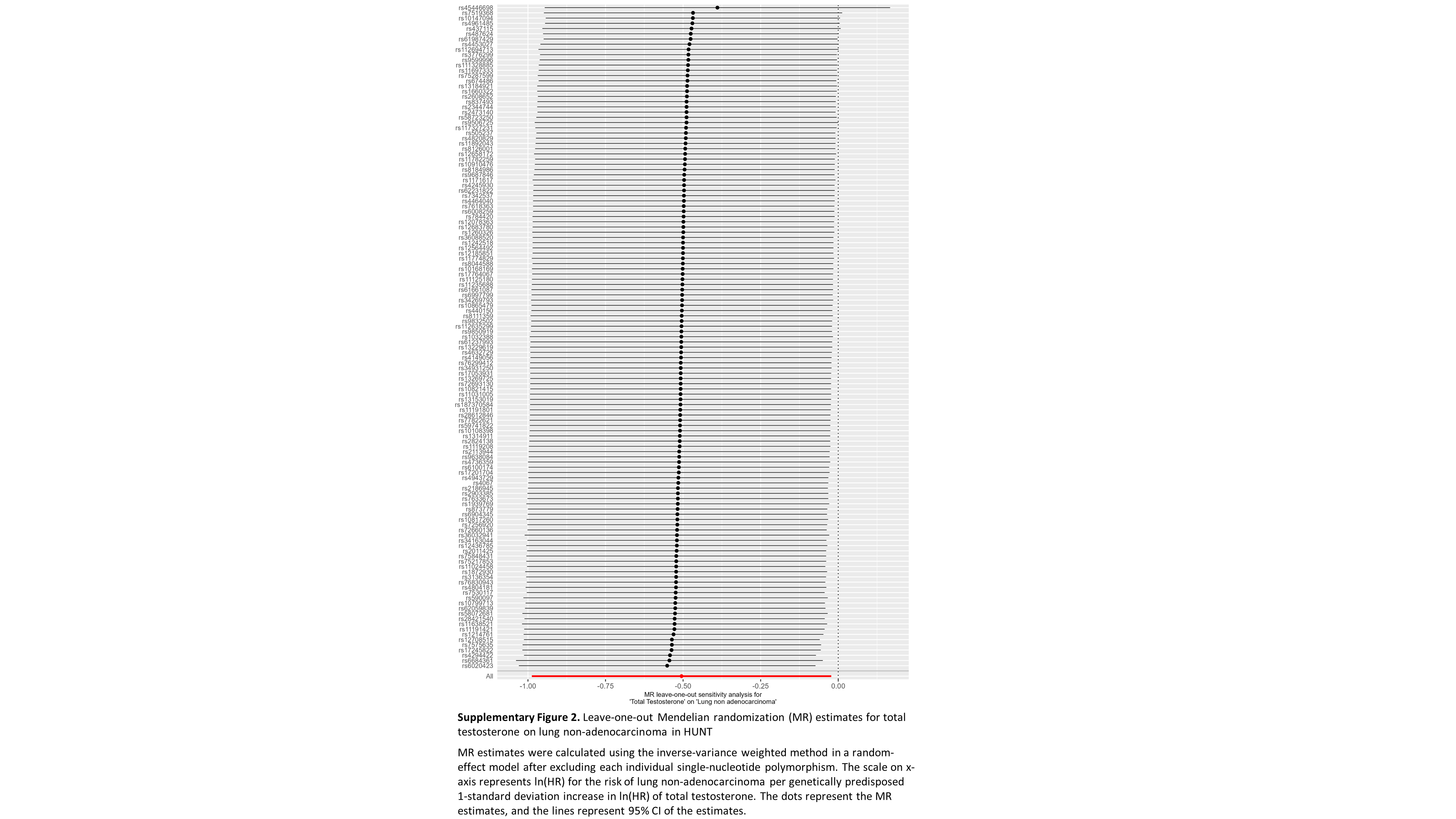

Supplement: Supplementary file 2 — Supplementary Material 2 [file 41598_2024_75305_MOESM2_ESM.tif]

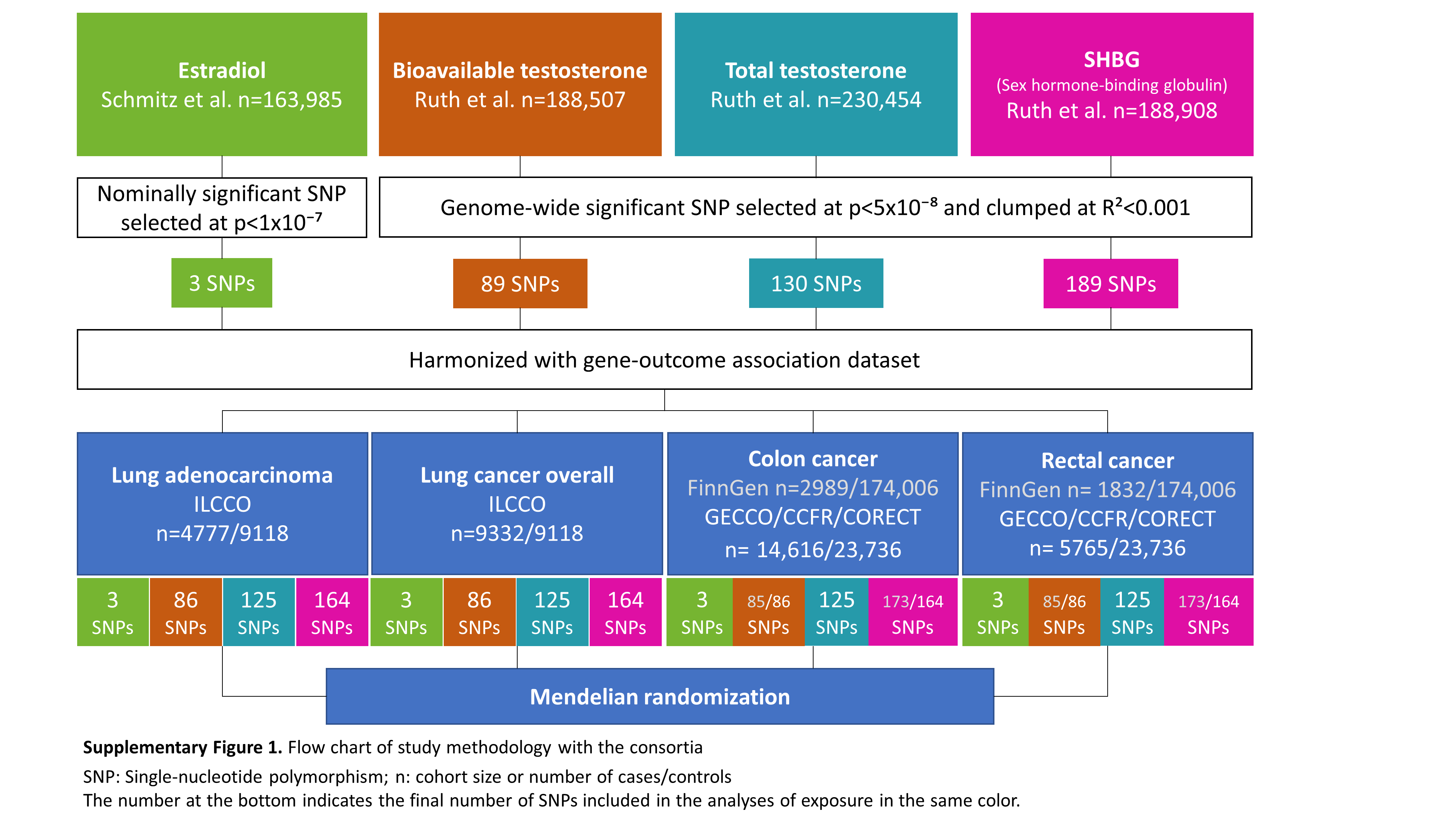

Supplement: Supplementary file 3 — Supplementary Material 3 [file 41598_2024_75305_MOESM3_ESM.tif]

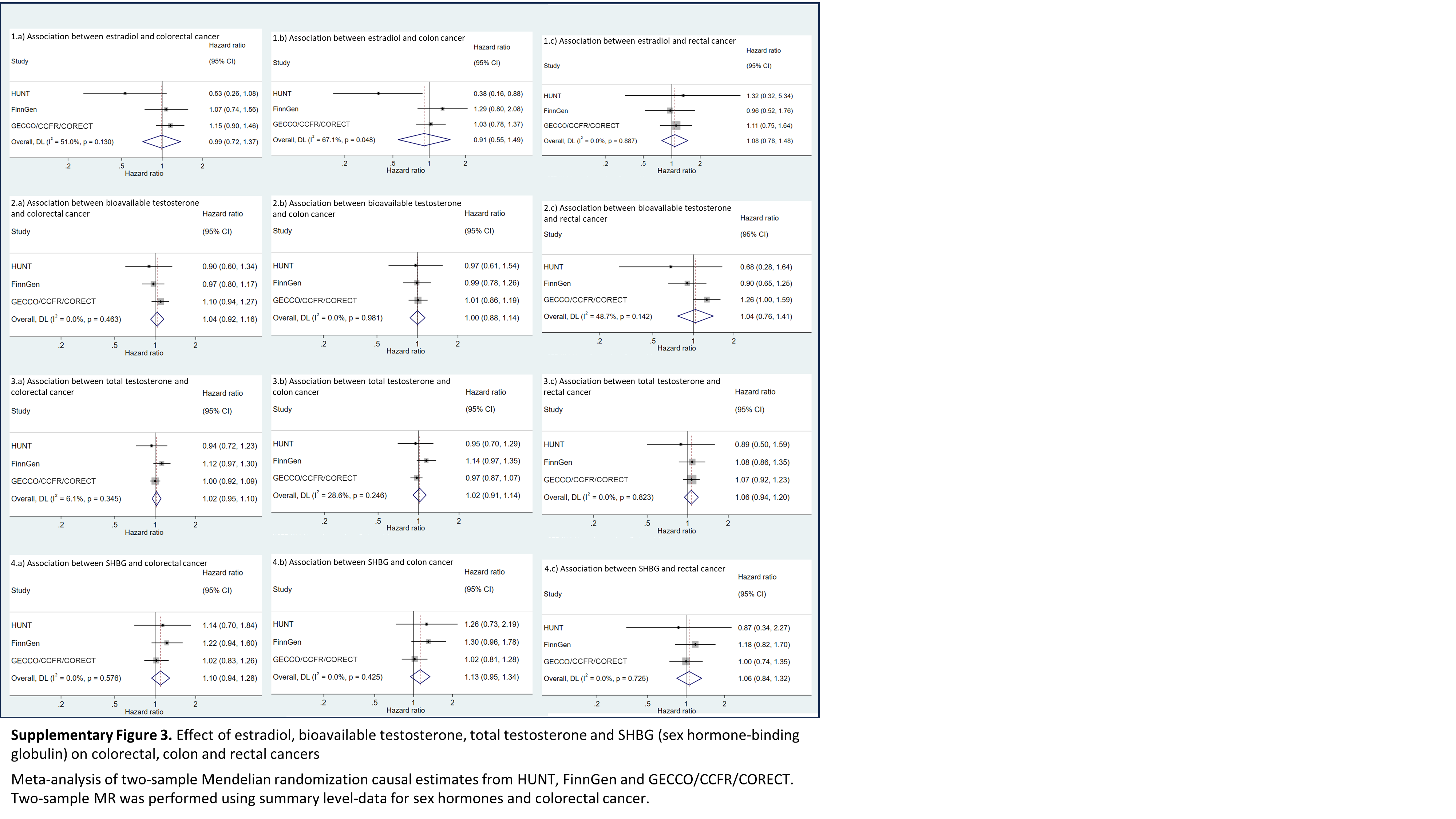

Supplement: Supplementary file 4 — Supplementary Material 4 [file 41598_2024_75305_MOESM4_ESM.tif]
